# Narrowing row spacing and adding inter-block promote the grain filling and flag leaf photosynthetic rate of wheat under enlarged drip tube spacing system

**DOI:** 10.3389/fpls.2024.1368410

**Published:** 2024-06-05

**Authors:** Jianguo Jing, Fu Qian, Xinyi Chang, Zhaofeng Li, Weihua Li

**Affiliations:** Key Laboratory of Oasis Eco-Agriculture, Xinjiang Production and Construction Corps, Agricultural College, Shihezi University, Shihezi, China

**Keywords:** wheat yield, drip tube configuration, grain weight, photosynthetic physiology, economic benefits

## Abstract

Enlarging the lateral space of drip tubes saves irrigation equipment costs (drip tubes and bypass), but it will lead to an increased risk of grain yield heterogeneity between wheat rows. Adjusting wheat row spacing is an effective cultivation measure to regulate a row’s yield heterogeneity. During a 2-year field experiment, we investigated the variations in yield traits and photosynthetic physiology by utilizing two different water- and fertilizer-demanding spring wheat cultivars (NS22 and NS44) under four kinds of drip irrigation patterns with different drip tube lateral spacing and wheat row spacing [① TR4, drip tube spacing (DTS) was 60 cm, wheat row horizontal spacing (WRHS) was 15 cm; ② TR6, DTS was 90 cm, WRHS was 15 cm; ③ TR6L, DTS was 90 cm, WRHS was 10 cm, inter-block spacing (IBS) was 35 cm; and ④ TR6S, DTS was 80 cm, WRHS was 10 cm, IBS was 25 cm]. The results showed that under 15-cm equal row spacing condition, after the number of wheat rows served by a single tube increased from four (TR4, control) to six (TR6), NS22 and NS44 exhibited a marked decline in yield. The decline of NS22 (9.93%) was higher than that of NS44 (9.04%), and both cultivars also showed a greater decrease in grain weight and average grain-filling rate (AGFR) of inferior grains (NS22: 23.19%, 13.97%; NS44: 7.78%, 5.86%) than the superior grains (NS22: 10.60%, 8.33%; NS44: 4.89%, 4.62%). After the TR6 was processed to narrow WRHS (from 15 to 10 cm) and add IBS (TR6L: 35 cm; TR6S: 25 cm), the grain weight per panicle (GWP) and AGFR of superior and inferior grains in the third wheat row (RW3) of NS22 and NS44 under TR6L increased significantly by 26.05%, 8.22%, 14.05%, 10.50%, 5.09%, and 5.01%, respectively, and under TR6S, they significantly increased by 20.78%, 9.91%, 16.19%, 9.28%, 5.01%, and 4.14%, respectively. The increase in GWP and AGFR was related to the increase in flag leaf area, net photosynthetic rate, chlorophyll content, relative water content, actual photochemical efficiency of PSII, and photochemical quenching coefficient. Among TR4, TR6, TR6L, and TR6S, for both NS22 and NS44, the yield of TR6S was significantly higher than that of TR6 and TR6L. Furthermore, TR6S showed the highest economic benefit.

## 1 Introduction

Wheat (*Triticum aestivum* L.) is one of the main food crops in the world (https://www.fao.org/faostat/en), with approximately 35% of the global population relying on wheat as their staple food (Li et al., 2019; Sultana et al., 2021), and has the characteristics of strong environmental adaptability and high nutritional value (Moreira-Ascarrunz et al., 2016; Liu et al., 2020; Song et al., 2021; Sheteiwy et al., 2022). Xinjiang’s wheat industry development level holds immense importance for both social stability and national food security as it serves as a prime region for producing superior quality wheat and acts as a crucial reserve base. However, due to Xinjiang’s location in the hinterland of the Eurasian continent, the annual rainfall is scarce, and the evaporation is huge. The frequency of drought and water shortage in wheat-producing areas has been on the rise (Kim and Jehanzaib, 2020), and will continue to increase in the future (Deng et al., 2006; Abdoulaye et al., 2019). The lack of water resources has become a key limiting factor to the development of wheat production (Gui et al., 2021; Zhang QL et al., 2021).

As an advanced water-saving irrigation technology, drip irrigation can simply, accurately, and stably transport a small amount of water to the roots of crops (Ma et al., 2020), with an irrigation efficiency as high as 75%–95% (Ward and Pulido-Velazquez, 2008). Applying drip irrigation technology to wheat production is the direction of agricultural development in Xinjiang and other arid agricultural areas of China (Wan et al., 2022). However, in recent years, the wheat area under drip irrigation in Xinjiang is approximately 0.3 million hm^2^ accounting for only a quarter of the wheat-planting area (Xinjiang Statistical Yearbook 2022). The direct reason for the difficulty in promoting wheat production application of drip irrigation technology is that the mainly popularized drip irrigation configuration in wheat production, which is one tube serving four rows of wheat (TR4), has the disadvantages of high drip tube and bypass consumption leading to high irrigation equipment cost. The indirect reason is that the entire drip irrigation systems is imitated from cash crop (cotton and corn) (Bozkurt et al., 2006). Furthermore, the underlying theoretical foundation is inadequate, and its mechanism research is not mature enough (Chen et al., 2015).

Wheat yield is determined by the number of panicles per unit area, the number of grains per panicle, and the grain weight (Guo LJ et al., 2021). As the final yield components, the formation of grain weight is influenced by cultivars, cultivation measures, and irrigation (Fischer, 2011; Watt et al., 2019; Zulfiqar et al., 2021). Grains per panicle are composed of superior and inferior grains (Yang et al., 2006). The superior grains have earlier flowering period, stronger ability to accumulate assimilates, and higher grain weight. However, the inferior grains have slower filling initiation, lower seed setting rate, and poorer fullness (Jiang et al., 2003; Yang and Zhang, 2010). Analyzing the variation of superior and inferior grain weight under different drip irrigation configurations [under different drip tube spacing (DTS) and wheat row horizontal spacing (WRHS)] can provide theoretical and technical support for optimizing wheat drip irrigation system. Previous studies have shown that soil water stress can reduce wheat yield by 20%–80%, which is mainly caused by a decrease in grain-filling rate and growth period (Foulkes et al., 2007; Li et al., 2011; Luo et al., 2019). Adequate soil moisture is beneficial to the demand of grain for carbon and nitrogen, which alleviates plant senescence. Moderate soil moisture deficiency enhances the re-transfer level of carbon and nitrogen stored in vegetative organs before flowering (Tiwari et al., 2021), and severe drought stress accelerates the loss of dry matter in leaves and aggravates plant senescence (Sharma and Dubey, 2005; Lafitte et al., 2006; Zhang et al., 2020). Photosynthesis is the basis for the synthesis and accumulation of organic matter in plants (Gautam et al., 2022; Zhang et al., 2021), and the photosynthetic carbon (C) assimilation of flag leaves during the grain-filling stage of wheat contributes the most carbohydrate substrates (>80%) for starch synthesis (Wu et al., 2012; Fan et al., 2017; Yang et al., 2022). The net photosynthetic (Pn) and transpiration rate (E) of crops vary with soil water content (Shemi et al., 2021). Under moderate irrigation conditions, the Pn of leaves always maintains a high level. On the contrary, under excessive irrigation or soil water-deficiency conditions, the Pn of leaves significantly decreases, and the photosynthetic function period is shortened (Anjum et al., 2011; Nezhadahmadi et al., 2013; Deng et al., 2018). Previous studies have shown that under sufficient water conditions, the Pn of wheat flag leaves begins to decline after 14 days of flowering (DAF). However, under soil water-deficit conditions, the Pn shows a rapid decline trend after 7 DAF (Liu et al., 2018; Zhu et al., 2020). The decrease in Pn is related to the decrease in chlorophyll content (Kocheva et al., 2004; Li et al., 2006) and photosynthetic system activity, which are caused by the inhibition of photosynthetic phosphorylation and electron transfer during the photosynthetic reaction process (Schmollinger et al., 2014). Recently, some scholars have proposed that enlarging DTS is one potential solution to reduce the drip tube use of wheat production under the drip irrigation system (Lv et al., 2019; Wan et al., 2022). However, under the enlarged drip irrigation system, the water supply in the soil of distant wheat row is less than that of the soil of adjacent row, which leads to growth differences in plant caused by spatial heterogeneity of soil water content (Li et al., 2004; Wang et al., 2006, 2013; Chen et al., 2015).

In the present research, two spring wheat cultivars (NS22, water and fertilizer demanding; NS44, water and fertilizer undemanding) were used as experimental materials. Through enlarging DTS (from 60 to 90 cm), narrowing WRHS (from 15 to 10 cm), and adding inter-block spacing (IBS; from 15 to 35 cm), four kinds of drip irrigation configurations were designed, namely: ① TR4 (a single tube serving four wheat rows, DTS was 60 cm, WRHS was 15 cm), ② TR6 (a single tube serving six wheat rows, DTS was 90 cm, WRHS was 15 cm), ③ TR6L (a single tube serving six wheat rows, DTS was 90 cm, WRHS was 10 cm, with large IBS: 35 cm), and ④ TR6S (a single tube serving six wheat rows, DTS was 80 cm, WRHS was 10 cm, with short IBS: 25 cm). We hypothesized that (H_1_) the superior and inferior grain weight and flag leaf photosynthetic physiological traits would be promoted by the changes in drip irrigation system, and (H_2_) we would also provide some constructive suggestions for further optimizing wheat drip irrigation system. The major objectives of this study were to focus on the following aspects: 1) to reveal the variations in superior and inferior grain weight, grain filling, and photosynthetic physiology among cultivars, configurations, and wheat rows of plants to explain the performances of grain yield and economic return, and 2) to further clarify the adaptation mechanism of wheat to drip irrigation. The results would provide theoretical and technical support for optimizing the drip irrigation system for wheat, and it would be possible to maximize the efficiency of the photosynthesis of wheat plant through artificial adjustment, which would contribute to food, resource, and environmental challenges encountered in Xinjiang and other arid agricultural regions.

## 2 Materials and methods

### 2.1 Research area detail

The experiment was conducted at a research station of Shihezi University, Xinjiang, in northwestern China (44°21′N, 86°04′E) from March to July. The area is situated at an elevation of 450 m, which experiences a typical temperate continental climate. The highest temperature is observed from July to early August, while the lowest temperature occurs in January. Additionally, the annual average precipitation ranges from 189.1 to 200.3 mm, while the annual potential evapotranspiration ranges from 1,517.5 to 1,563.8 mm. **Supplementary Figure 1** displays the daily precipitation and maximum/minimum air temperature throughout the experimental period. The experimental site has been planted with wheat for many years, and a season of cabbage was planted every year after the end of the experiment (July to October). The soil at the experiment farm has moderate fertility, and the physical and chemical properties of the soil profile (average of 2 years) at the experimental site before sowing are shown in **Table 1**.

**Table 1:** Physical and chemical properties of the soil profile before sowing (0–60 cm depth).

Parameter	Average
pH	7.6 ± 0.2
Organic matter (g kg^−1^)	11.4 ± 2.5
Alkaline-N (mg kg^−1^)	42.1 ± 1.2
Olsen-P (mg kg^−1^)	13.8 ± 0.6
Available K (mg kg^−1^)	295.0 ± 4.6
Bulk density (g cm^−3^)	1.2 ± 0.8

### 2.2 Planting material

Spring wheat, *cv* New Spring22 (NS22) and New Spring44 (NS44), which are widely planted spring wheat varieties in Xinjiang, were sown at a rate of 600 × 10^4^ plant ha^–2^ (The photos of NS22 and NS44 growth under TR4, TR6, TR6L, and TR6S configurations in the field are shown in **Supplementary Figure 2**). The sowing dates were 27 March 2020 and 9 April 2021. In preliminary cultivar screening experiments (20 varieties) under the enlarged DTS system, we found that NS22 (12.78) showed the highest coefficient of variation between row yield, and NS44 (3.31) showed the lowest coefficient of variation between row yield. Thus, we defined NS22 as a water- and fertilizer-demanding cultivar and NS44 as a water- and fertilizer-undemanding cultivar (Yang JP et al., 2020). The difference in flowering and growth periods between NS22 and NS44 were 2–3 days and 9–11 days, respectively.

### 2.3 Experimental design

**Figure 1:**
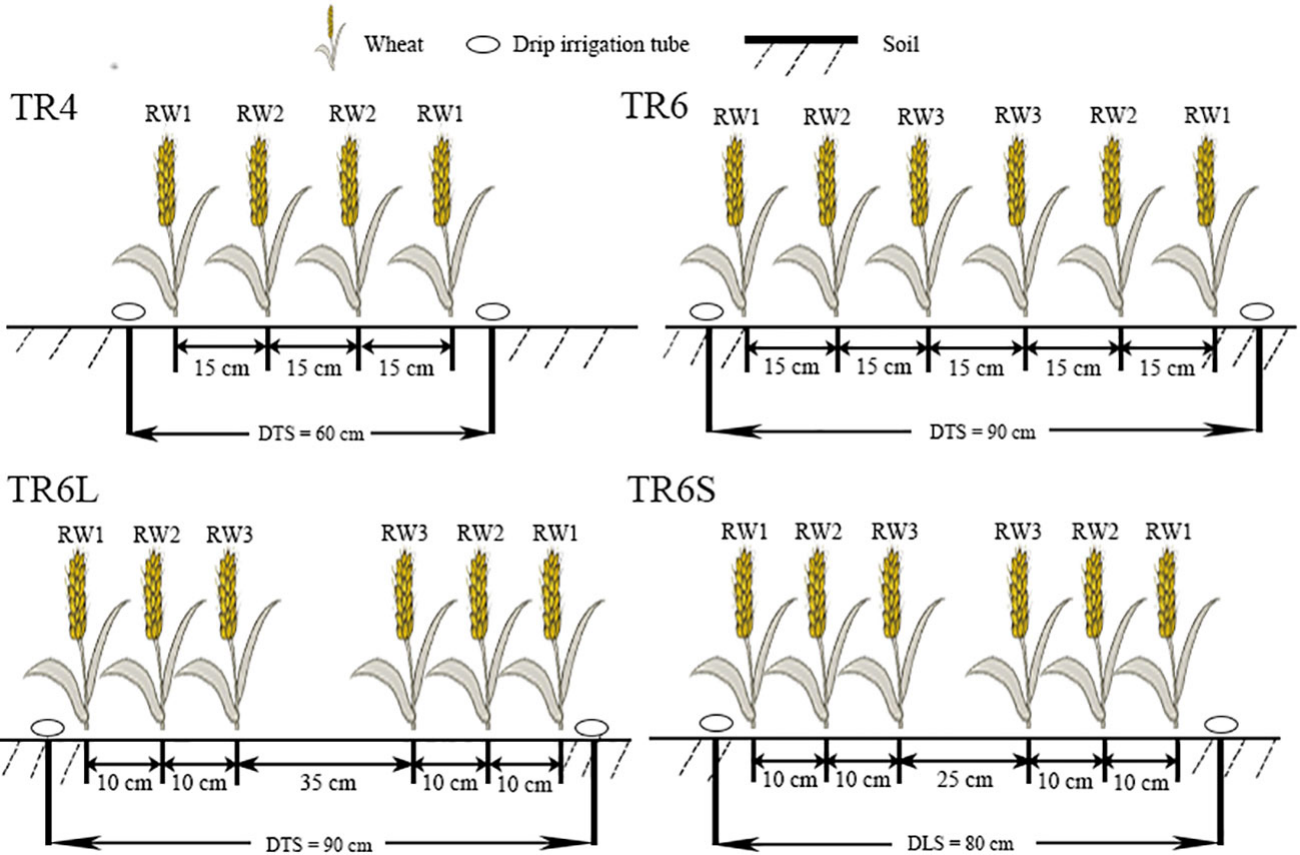
Schematic diagram of TR4 [drip tube spacing (DTS) was 60 cm, wheat row horizontal spacing (WRHS) was 15 cm], TR6 (DTS was 90 cm, WRHS was 15 cm), TR6L (DTS was 90 cm, WRHS was 10 cm, IBS was 35 cm), and TR6S (DTS was 80 cm, WRHS was 10 cm, IBS was 25 cm). RW1, RW2, and RW3 represent the first, second, and third rows of wheat plants close to the drip tube, respectively.

The experiment designed four kinds of drip irrigation configurations, namely: ① TR4 (as control, a single tube serving four wheat rows, which is extensively employed in wheat production in Xinjiang, DTS was 60 cm, WRHS was 15 cm), ② TR6 (a single tube serving six wheat rows, DTS was 90 cm, WRHS was 15 cm), ③ TR6L (a single tube serving six wheat rows, DTS was 90 cm, WRHS was 10 cm, with large IBS: 35 cm), and ④ TR6S (a single tube serving six wheat rows, DTS was 80 cm, WRHS was 10 cm, with short IBS: 25 cm). Each configuration was replicated three times. The schematic diagram of the four kinds of drip irrigation configurations are shown in **Figure 1**. RW1, RW2, and RW3 represent the first, second, and third rows of wheat plants close to the drip tube, respectively. Twelve plots were arranged in a randomized block design, separated from adjacent plots by approximately 1-m-wide isolation strips. The plots for TR4, TR6, TR6L, and TR6S were 7.2 × 27 m (194.4 m^2^), 7.2 × 27 m (194.4 m^2^), 7.2 × 27 m (194.4 m^2^), and 6.4 × 27 m (172.8 m^2^), respectively. The drip irrigation configurations in 2020 and 2021 were the same. The positioning of synchronous drip tubes and sowing were carried out using precision planters specifically designed for wheat (JB/T 6274.1–2013, 2BFX-12, China). The sowing depth was approximately 5.0 cm, and the drip tube covered a depth of approximately 2 cm. The outlet holes of the drip tube were designed in a single-wing maze with a spacing of 30 cm and a flow rate of 2.6 L h^−1^. Total irrigation volume and urea content of each plot were 45,00 m^3^ ha^−1^ and 300 kg ha^−1^, respectively. The timing and volume of irrigation and fertilization followed those of previous studies (Liu et al., 2013; Lv et al., 2019). In brief, the irrigation amount for the three-leaf stage, jointing, booting, anthesis, early milk stage, and late milk stage were 900, 900, 900, 675, 675, and 450 m^3^ ha^−1^, respectively. The nitrogen application amount for pre-sowing, three-leaf stage, jointing, booting, anthesis, and early milk stage were 60, 36, 96, 48, 36, and 24 kg ha^−1^, respectively. To facilitate precise measurement and control, each experimental plot was connected to a high-precision water meter and control valve. Additionally, before sowing, 105 kg ha^−2^ of P_2_O_5_ and K_2_O was applied to enrich the soil nutrient content.

### 2.4 Sampling and measurements

At the seedling stage, 10 representative squares for each plot were selected and marked (TR4, TR6, TR6L, and TR6S square sizes were 0.6, 0.9, 0.9, and 0.8 m^2^, respectively). For each square, 50 plants with the same flowering date were selected and marked with wool. In the marked square, sampling and index measurement in a single row (TR4: RW1 and RW2, TR6, TR6L and TR6S: RW1, RW2, and RW3) were taken at 5-day intervals from 7 days after flowering (DAF) to maturity.

#### 2.4.1 Yield, yield components, and economic benefit

At maturity, 20 wheat plants were randomly and then consecutively selected from each row to determine superior and inferior grain weight per panicle; three representative squares of each plot were selected to measure the panicle number and yield of each row. Of each row, 1,000 grains were randomly selected to test the thousand grain weight (TGW) repeating three times. Economic benefit was calculated using Equation 1:


(1) $$\begin{array}{c} \text{Economic benefit }\left(\text{US}\${\text{ ha}}^{-1}\right)=\text{grain yield }\left({\text{kg ha}}^{-1}\right)\\ \times \text{wheat price }\left(\text{US}\${\text{ kg}}^{-1}\right)-\text{seed cost }\left(\text{US}\${\text{ ha}}^{-1}\right)\\ -\text{irrigation equipment cost }\left(\text{US}\${\text{ ha}}^{-1}\right)\\ -\text{ fertilizer cost }\left(\text{US}\${\text{ ha}}^{-1}\right)-\text{water cost }\left({\text{m}}^{3}{\text{ha}}^{-1}\right)-\text{pesticide cost }\left(\text{US}\${\text{ ha}}^{-1}\right) \end{array}$$

#### 2.4.2 Grain-filling process

Samples were taken at 7, 13, 19, 25, and 31 DAF, and grains were divided into superior and inferior grains according to the classification method (Jiang et al., 2003). All samples were fixed for 30 min at 105°C, dried, and weighed at 70°C. The growth period = the date of death of wheat plants (more than 50%) − the date of emergence of wheat plants (more than 50%).

#### 2.4.3 Gas exchange

At 7, 13, 19, 25, and 31 DAF, the flag leaves with the same growth were selected to measure gas exchange parameters. The Pn, stomatal conductance (gs), intercellular CO_2_ concentration (Ci), and E were measured from 10–12 h using a Portable Photosynthesis System (Li-6400, Li-COR Inc., NE, USA) at a light intensity of 1,700 μmol (photon) m^–2^ s^–1^ under uniform conditions [25–32°C, 400–500 μmol (CO_2_) mol^–1^] according to Jing et al. (2019). Nine flag leaves were determined for each treatment.

#### 2.4.4 Chlorophyll content

At 7, 13, 19, 25, and 31 DAF, the chlorophyll content (CC) in the flag leaves was determined by a Portable Chlorophyll content meter (SPAD502, Litai, Japan). Fifteen leaves were measured for each row, and each leaf was measured three times by dividing it into leaf tip, middle leaf, and leaf base. The average value was taken as the SPAD value of a leaf.

#### 2.4.5 Relative water content and area of flag leaf

The flag leaves were taken at 7, 13, 19, 25, and 31 DAF. The flag leaves were washed clean, and the surface water was absorbed by filter paper and weighed, with the weight considered as the fresh weight. The samples were put into an oven at a temperature of 105°C for a duration of 30 min, subsequently drying them at 70°C until a constant weight is achieved, and then weighed, with the weight representing the dry weight. Relative water content (RWC) was calculated using Equation 2:


(2) Water content (%)=[fresh weight (g)−dry weight (g)]/fresh weight (g)×100

The flag leaf area (FLA) was determined by area meter (LA211, Systronic, New Delhi, India). Fifteen leaves were measured for each row.

#### 2.4.6 Chlorophyll fluorescence

At 7, 13, 19, 25, and 31 DAF, the same leaves employed for the gas exchange measurement were used to determine chlorophyll fluorescence using a portable fluorometer (PAM-2100, Walz, Germany). The fluorescence kinetic parameters were calculated according to the method of previous researchers (Genty et al., 1989).

### 2.5 Statistical analysis

One-way analysis of variance was applied to determine differences among treatments. The results were described as the means of three replicates ± SD. Data were analyzed using a statistical package (ANOVA v. 2017. Nanjing Agricultural University, Nanjing, China). Mean values were compared by applying Duncan’s multiple range test at the 0.05 level of significance. Path analysis was conducted using linear regression analysis from SPSS v. 25 (SPSS Inc., Chicago, Illinois, USA). Microsoft Excel (Office v. 2010) and Origin v. 2021 (Origin Lab, Northampton, MA, USA) were utilized for data collation and to plot figures.

## 3 Results

### 3.1 Yield and yield components

Among TR4, TR6, TR6L, and TR6S, both NS22 and NS44 in 2020 and 2021 showed the highest yield in TR4 (**Table 2**) and the highest economic benefits in TR6S. Compared with TR4, the yield decrease in TR6, TR6L, and TR6S of NS44 (8.84%, 6.16%, and 1.79%, respectively) was lower than that of NS22 (10.56%, 10.89%, and 3.59%, respectively). The grain weight per panicle (GWP) decrease of NS44 (5.76%, 4.25%, and 5.72%) was also lower than that of NS22 (13.95%, 8.10%, and 9.79%), while the thousand grain weight (TGW) decrease of NS44 (6.62%, 4.95%, and 4.10%) was higher than that of NS22 (3.55%, 4.23%, and 1.60%). The GWP in superior grains of NS22 and NS44 decreased by 3.49%–10.60% and that of inferior grains decreased by 6.02%–23.19%. Compared with TR4, for both NS22 and NS44, TR6S showed the largest decrease in panicle number among TR6, TR6L, and TR6S, with a decrease of 8.97% and 5.96%, respectively. However, TR6S also showed the lowest decrease in yield indicating that the yield decrease of TR6S might be related to the decrease in panicle number.

**Table 2:** Overall yield, weight per panicle (GWP), panicle number, thousand grain weight (TGW), total cost, and economic benefit of two cultivars (NS22 and NS44) under four kinds of drip irrigation configurations.

Year	Treatment	Weight per panicle of superior grains (g)	Weight per panicle of inferior grains (g)	Weight per panicle (g)	Panicle number	Thousand grains weight (g)	Grain yield (kg ha^−2^)	Total cost (US$ ha^−1^)	Economic benefit (US$ ha^−1^)
2020	ATR4	1.14 ± 0.008^d^	0.41 ± 0.006^c^	1.55 ± 0.004^d^	107.33 ± 0.441^a^	44.09 ± 0.430^d^	7,780.31 ± 52.671^b^	988.77	2,037.03 ± 2.197^e^
	ATR6	1.02 ± 0.007^g^	0.32 ± 0.009^e^	1.33 ± 0.004^g^	100.04 ± 0.706^c^	42.52 ± 0.158^f^	7,007.63 ± 95.166^d^	826.48	1,886.98 ± 7.233^g^
	ATR6L	1.07 ± 0.005^e^	0.35 ± 0.004^d^	1.42 ± 0.001^e^	103.37 ± 0.849^b^	42.22 ± 0.138^f^	7,011.93 ± 58.350^d^	826.48	1,926.19 ± 7.778^f^
	ATR6S	1.06 ± 0.009^f^	0.34 ± 0.007^d^	1.40 ± 0.005^f^	97.70 ± 0.804^d^	43.38 ± 0.243^e^	7,515.31 ± 59.609^c^	867.05	2,101.82 ± 8.830^d^
	BTR4	1.30 ± 0.005^a^	0.56 ± 0.011^a^	1.86 ± 0.007^a^	88.89 ± 1.182^f^	53.37 ± 0.314^a^	8,268.58 ± 31.477^a^	988.77	2,211.50 ± 6.138^c^
	BTR6	1.24 ± 0.007^c^	0.51 ± 0.004^b^	1.75 ± 0.004^c^	88.93 ± 0.170^f^	49.84 ± 0.571^c^	7,521.41 ± 17.908^c^	826.48	2,091.86 ± 7.264^d^
	BTR6L	1.25 ± 0.003^b^	0.52 ± 0.004^b^	1.78 ± 0.007^b^	90.48 ± 0.280^e^	50.73 ± 0.206^b^	7,826.00 ± 63.472^b^	826.48	2,245.71 ± 12.132^b^
	BTR6S	1.23 ± 0.004^c^	0.52 ± 0.001^b^	1.75 ± 0.003^c^	83.59 ± 0.231^g^	51.18 ± 0.222^b^	8,182.25 ± 30.936^a^	867.05	2,332.44 ± 9.620^a^
2021	ATR4	1.12 ± 0.015^d^	0.39 ± 0.003^c^	1.51 ± 0.013^d^	107.11 ± 1.084^a^	43.82 ± 0.395^e^	7,373.67 ± 74.514^c^	988.77	1,873.27 ± 2.728^e^
	ATR6	1.00 ± 0.008^f^	0.31 ± 0.005^f^	1.32 ± 0.012^g^	99.70 ± 0.612^c^	42.12 ± 0.078^g^	6,548.46 ± 59.120^f^	826.48	1,715.65 ± 11.843^f^
	ATR6L	1.06 ± 0.005^e^	0.34 ± 0.001^d^	1.40 ± 0.004^e^	103.67 ± 0.484^b^	41.74 ± 0.126^g^	6,496.52 ± 67.292^f^	826.48	1,722.58 ± 7.689^f^
	ATR6S	1.04 ± 0.007^e^	0.33 ± 0.004^e^	1.38 ± 0.006^f^	97.70 ± 0.170^d^	42.84 ± 0.330^f^	7,095.44 ± 89.386^e^	867.05	1,944.96 ± 6.473^d^
	BTR4	1.26 ± 0.007^a^	0.53 ± 0.011^a^	1.79 ± 0.004^a^	90.28 ± 1.262^e^	52.83 ± 0.008^a^	7,883.47 ± 132.706^a^	988.77	2,048.48 ± 8.223^b^
	BTR6	1.20 ± 0.008^c^	0.48 ± 0.004^b^	1.68 ± 0.006^c^	88.44 ± 0.294^f^	49.44 ± 0.617^d^	7,203.07 ± 7.994^de^	826.48	1,976.53 ± 6.177^c^
	BTR6L	1.22 ± 0.006^b^	0.49 ± 0.005^b^	1.71 ± 0.009^b^	90.44 ± 0.111^e^	50.44 ± 0.125^c^	7,334.41 ± 48.759^cd^	826.48	2,052.19 ± 12.769^b^
	BTR6S	1.20 ± 0.005^c^	0.49 ± 0.002^b^	1.69 ± 0.003^c^	83.04 ± 0.390^g^	51.30 ± 0.360^b^	7,683.65 ± 53.900^b^	867.05	2,153.81 ± 2.693^a^

A and B represent NS22 and NS44, respectively. TR4 represents a single tube serving four wheat rows under equal row spacing planting, WRHS was 15 cm and DTS was 60 cm; TR6 represents a common single tube serving six wheat rows under equal row spacing planting, DTS was 90 cm, WRHS was 15 cm; TR6L represents an enlarged single tube serving six wheat rows under wide–narrow row planting, DTS was 90 cm, WRHS was 10 cm, IBS was 35 cm; TR6S represents a shortened single tube serving six wheat rows under wide–narrow row planting, DTS was 80 cm, WRHS was 10 cm, IBS was 25 cm. The prices of wheat grain, single bypass, and drip tube were 0.39 US$ kg^−1^, 0.04 US$, and 0.028 US$ m^−1^, respectively. Other costs included water consumption (180 US$ ha^−1^), seed (165.3 US$ ha^−1^), and fertilizers and pesticides (155.6 US$ ha^−1^). All the values are average of three replicates. Means that do not share the same letters in the column differ obviously at p < 0.05.

In 2020 and 2021, under TR4, the GWP, panicle number, TGW, and yield in RW1 of both NS22 and NS44 were not significantly different from those in RW2 (**Table 3**), while after increasing the DTS from 60 to 90 cm (TR6), the yield and GWP of both NS22 and NS44 showed RW3 < RW2 < RW1, and the differences were significant; TGW in RW3 of NS22 was significantly higher than that of RW1, while NS44 showed that RW3 was not significantly different from RW1 and RW2. After the TR6 was processed to narrow WRHS and add IBS (TR6L and TR6S), the GWP and yield in RW3 of NS22 (GWP: 26.05% and 20.78%; yield: 19.64% and 30.02%) and NS44 (10.50%, 9.28%, 20.35%, and 26.75%) significantly increased; however, those in RW1 decreased by 2.84%–16.59%. Under TR6L and TR6S, the TGW of RW3 was not significantly different from that of TR4RW1 and TR4RW2, while the GWP and IGWP significantly decreased, which indicated that the decrease in GWP of RW3 may be related to the decrease in inferior grain number.

**Table 3:** Variations in yield, weight per panicle (GWP), panicle number, and thousand grains weight (TGW) of two cultivars (NS22 and NS44) under four kinds of drip irrigation configurations.

Year	Treatment	Weight per panicle of superior grains (g)	Weight per panicle of inferior grains (g)	Weight per panicle (g)	Panicle number	Thousand grains weight (g)	Grain yield (kg ha^−2^)
2020	ATR4RW1	1.14 ± 0.005^h^	0.41 ± 0.002^e^	1.55 ± 0.005^h^	107.56 ± 1.171^a^	43.89 ± 0.429^hi^	3,892.00 ± 31.177^b^
	ATR4RW2	1.14 ± 0.012^h^	0.41 ± 0.012^e^	1.55 ± 0.006^h^	107.11 ± 0.694^a^	44.29 ± 0.441^gh^	3,888.31 ± 24.939^b^
	ATR6RW1	1.09 ± 0.005^j^	0.38 ± 0.005^f^	1.47 ± 0.001^j^	102.44 ± 1.171^c^	41.81 ± 0.165^l^	2,563.11 ± 23.041^fg^
	ATR6RW2	1.03 ± 0.009^m^	0.32 ± 0.015^i^	1.35 ± 0.01^n^	104.44 ± 1.347^b^	42.56 ± 0.205^k^	2,352.93 ± 52.224^h^
	ATR6RW3	0.93 ± 0.008^n^	0.25 ± 0.008^j^	1.18 ± 0.001°	93.22 ± 1.018^fg^	43.20 ± 0.270^ijk^	2,091.59 ± 67.293^j^
	ATR6LRW1	1.05 ± 0.006^l^	0.34 ± 0.004^ghi^	1.38 ± 0.003^m^	100.67 ± 1.764^d^	40.14 ± 0.258^m^	2,137.87 ± 33.595^j^
	ATR6LRW2	1.06 ± 0.002^kl^	0.34 ± 0.004^gh^	1.40 ± 0.002^l^	103.33 ± 0.333^bc^	41.78 ± 0.307^l^	2,371.78 ± 49.719^h^
	ATR6LRW3	1.11 ± 0.013^i^	0.38 ± 0.010^f^	1.49 ± 0.002^i^	106.11 ± 0.509^a^	44.74 ± 0.536^g^	2,502.28 ± 37.288^g^
	ATR6SRW1	1.05 ± 0.009^l^	0.33 ± 0.007^hi^	1.38 ± 0.005^m^	93.67 ± 0.882^ef^	42.70 ± 0.271^jk^	2,382.71 ± 69.743^h^
	ATR6SRW2	1.05 ± 0.009^l^	0.34 ± 0.006^gh^	1.39 ± 0.007^m^	95.11 ± 0.694^e^	43.32 ± 0.197^ij^	2,413.02 ± 17.529^h^
	ATR6SRW3	1.07 ± 0.012^jk^	0.35 ± 0.009^g^	1.42 ± 0.004^k^	104.33 ± 0.882^b^	44.13 ± 0.318^gh^	2,719.58 ± 65.471^d^
	BTR4RW1	1.30 ± 0.007^a^	0.56 ± 0.011^a^	1.86 ± 0.018^a^	89.00 ± 1.453^j^	53.30 ± 0.377^a^	4,134.61 ± 74.512^a^
	BTR4RW2	1.30 ± 0.013^ab^	0.56 ± 0.013^a^	1.86 ± 0.007^a^	88.78 ± 1.018^j^	53.45 ± 0.472^a^	4,133.97 ± 49.432^a^
	BTR6RW1	1.28 ± 0.006^bc^	0.54 ± 0.008^b^	1.82 ± 0.004^b^	89.89 ± 0.694^ij^	49.64 ± 1.087^de^	2,683.24 ± 4 8.296^de^
	BTR6RW2	1.26 ± 0.009^de^	0.52 ± 0.006^c^	1.78 ± 0.009^d^	91.11 ± 0.509^hi^	49.65 ± 0.690^de^	2,602.39 ± 21.217^f^
	BTR6RW3	1.16 ± 0.009^g^	0.48 ± 0.002^d^	1.65 ± 0.010^g^	85.78 ± 0.694^k^	50.23 ± 0.219^cd^	2,235.78 ± 14.133^i^
	BTR6LRW1	1.25 ± 0.012^e^	0.52 ± 0.008^c^	1.77 ± 0.013^d^	89.89 ± 0.694^ij^	48.91 ± 0.427^f^	2,589.91 ± 32.377^f^
	BTR6LRW2	1.23 ± 0.008^f^	0.52 ± 0.010^c^	1.74 ± 0.008^e^	89.44 ± 0.509^j^	50.47 ± 0.324^c^	2,545.28 ± 43.688^fg^
	BTR6LRW3	1.28 ± 0.009^bc^	0.54 ± 0.005^b^	1.82 ± 0.005^b^	92.11 ± 0.694^gh^	52.80 ± 0.123^a^	2,690.81 ± 49.458^d^
	BTR6SRW1	1.22 ± 0.013^f^	0.51 ± 0.008^c^	1.73 ± 0.006^f^	81.00 ± 0.667^m^	48.97 ± 0.350^ef^	2,609.29 ± 29.671^ef^
	BTR6SRW2	1.21 ± 0.008^f^	0.51 ± 0.007^c^	1.72 ± 0.002^f^	83.11 ± 0.509^l^	51.50 ± 0.396^b^	2,739.06 ± 17.957^d^
	BTR6SRW3	1.27 ± 0.016^cd^	0.53 ± 0.010^bc^	1.80 ± 0.007^c^	86.67 ± 0.882^k^	53.07 ± 0.235^a^	2,833.90 ± 39.052^c^
2021	ATR4RW1	1.12 ± 0.012^e^	0.39 ± 0.003^f^	1.51 ± 0.011^h^	107.22 ± 1.836^a^	43.48 ± 0.168^gh^	3,697.47 ± 30.683^b^
	ATR4RW2	1.12 ± 0.021^e^	0.39 ± 0.004^f^	1.51 ± 0.020^h^	107.00 ± 0.333^a^	44.16 ± 0.629^fg^	3,676.19 ± 44.659^b^
	ATR6RW1	1.09 ± 0.007^fg^	0.37 ± 0.012^g^	1.46 ± 0.015^i^	101.89 ± 1.262^c^	41.31 ± 0.236^i^	2,395.52 ± 24.854^fg^
	ATR6RW2	1.02 ± 0.010^j^	0.32 ± 0.006^j^	1.33 ± 0.011^m^	104.78 ± 0.509^b^	41.96 ± 0.208^i^	2,211.02 ± 23.153^hi^
	ATR6RW3	0.91 ± 0.015^k^	0.24 ± 0.004^k^	1.16 ± 0.010^n^	92.44 ± 1.388^fg^	43.08 ± 0.220^h^	1,941.93 ± 61.278^j^
	ATR6LRW1	1.03 ± 0.009^ij^	0.33 ± 0.013^hij^	1.36 ± 0.009^l^	99.78 ± 1.644^d^	39.65 ± 0.088^j^	1,977.09 ± 26.372^j^
	ATR6LRW2	1.05 ± 0.014^hi^	0.34 ± 0.005^hi^	1.39 ± 0.017^jk^	103.44 ± 2.589^bc^	41.25 ± 0.304^i^	2,187.69 ± 34.521^hi^
	ATR6LRW3	1.09 ± 0.021^f^	0.37 ± 0.016^g^	1.46 ± 0.027^i^	107.78 ± 0.839^a^	44.32 ± 0.221^f^	2,331.74 ± 30.616^g^
	ATR6SRW1	1.03 ± 0.022^j^	0.32 ± 0.010^ij^	1.35 ± 0.013^lm^	93.67 ± 0.333^ef^	41.94 ± 0.371^i^	2,215.13 ± 47.847^h^
	ATR6SRW2	1.04 ± 0.005^ij^	0.34 ± 0.001^hi^	1.37 ± 0.006^kl^	95.00 ± 0.333^e^	43.08 ± 0.556^h^	2,348.23 ± 37.335^fg^
	ATR6SRW3	1.07 ± 0.006^gh^	0.34 ± 0.006^h^	1.41 ± 0.006^j^	104.44 ± 0.509^b^	43.52 ± 0.162^gh^	2,532.08 ± 92.266^d^
	BTR4RW1	1.27 ± 0.009^a^	0.53 ± 0.009^a^	1.79 ± 0.013^a^	90.00 ± 1.453^hi^	52.73 ± 0.231^a^	3,941.72 ± 80.733^a^
	BTR4RW2	1.26 ± 0.014^a^	0.53 ± 0.012^a^	1.79 ± 0.006^a^	90.56 ± 1.836^ghi^	52.93 ± 0.237^a^	3,941.75 ± 56.377^a^
	BTR6RW1	1.25 ± 0.007^ab^	0.51 ± 0.007^b^	1.76 ± 0.011^b^	89.67 ± 1.000^i^	49.44 ± 1.029^cd^	2,553.02 ± 15.826^d^
	BTR6RW2	1.23 ± 0.011^bc^	0.49 ± 0.008^cd^	1.72 ± 0.015^de^	90.11 ± 0.694^hi^	49.07 ± 0.947^de^	2,512.43 ± 18.345^d^
	BTR6RW3	1.13 ± 0.012^e^	0.45 ± 0.005^e^	1.58 ± 0.016^g^	85.56 ± 0.509^j^	49.81 ± 0.380^cd^	2,137.63 ± 11.813^i^
	BTR6LRW1	1.22 ± 0.009^c^	0.49 ± 0.006^cd^	1.70 ± 0.011^e^	89.67 ± 0.333^i^	48.50 ± 0.488^e^	2,422.72 ± 32.794^ef^
	BTR6LRW2	1.19 ± 0.011^d^	0.49 ± 0.011^cd^	1.68 ± 0.021^f^	89.78 ± 0.839^i^	50.26 ± 0.042^c^	2,380.13 ± 41.682^fg^
	BTR6LRW3	1.25 ± 0.011^ab^	0.50 ± 0.009^bc^	1.75 ± 0.003^bc^	91.89 ± 0.509^fgh^	52.56 ± 0.147^a^	2,531.56 ± 37.118^d^
	BTR6SRW1	1.18 ± 0.010^d^	0.48 ± 0.011^d^	1.67 ± 0.002^f^	80.67 ± 0.667^l^	49.77 ± 0.879^cd^	24,85.69 ± 14.875^de^
	BTR6SRW2	1.18 ± 0.012^d^	0.48 ± 0.007^d^	1.66 ± 0.007^f^	82.56 ± 0.694^k^	51.55 ± 0.344^b^	2,551.56 ± 17.957^d^
	BTR6SRW3	1.23 ± 0.019^bc^	0.50 ± 0.013^bcd^	1.73 ± 0.008^cd^	85.89 ± 0.694^j^	52.58 ± 0.155^a^	2,646.40 ± 39.052^c^

A and B represent NS22 and NS44, respectively. TR4 represents a single tube serving four wheat rows under equal row spacing planting, WRHS was 15 cm and DTS was 60 cm; TR6 represents a common single tube serving six wheat rows under equal row spacing planting, DTS was 90 cm, WRHS was 15 cm; TR6L represents an enlarged single tube serving six wheat rows under wide–narrow row planting, DTS was 90 cm, WRHS was 10 cm, IBS was 35 cm; TR6S represents a shortened single tube serving six wheat rows under wide–narrow row planting, DTS was 80 cm, WRHS was 10 cm, IBS was 25 cm. RW1, RW2, and RW3 represent the first, second, and third rows of wheat plants close to the drip tube, respectively. All the values are average of three replicates. Means that do not share the same letters in the column differ obviously at p < 0.05.

### 3.2 Grain filling

**Figure 2:**
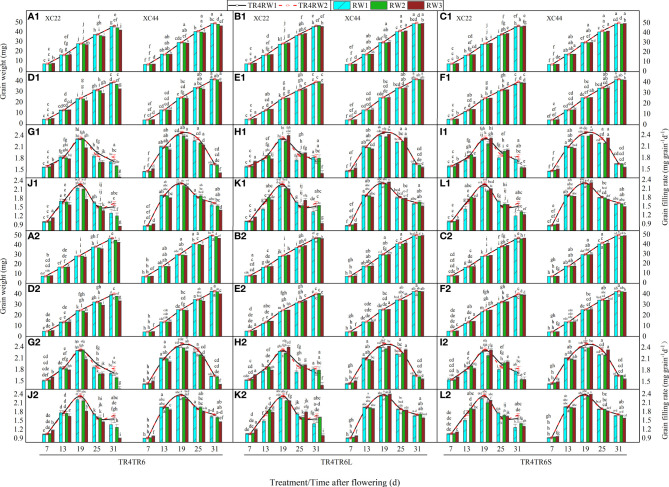
Changes in grain weight and grain-filling rate with the days after flowering of two cultivars (NS22 and NS44) under four kinds of drip irrigation configurations in 2020 and 2021. **(A–C, G–I)** represent superior grain; **(D–F, J–L)** represent inferior grain. TR4 represents a single tube serving four wheat rows under equal row spacing planting, WRHS was 15 cm and DTS was 60 cm; TR6 represents a common single tube serving six wheat rows under equal row spacing planting, DTS was 90 cm, WRHS was 15 cm; TR6L represents an enlarged single tube serving six wheat rows under wide–narrow row planting, DTS was 90 cm, WRHS was 10 cm, IBS was 35 cm; TR6S represents a shortened single tube serving six wheat rows under wide–narrow row planting, DTS was 80 cm, WRHS was 10 cm, IBS was 25 cm. 1 indicates 2020; 2 indicates 2021. RW1, RW2, and RW3 represent the first, second, and third rows of wheat plants close to the drip tube, respectively. All the values are average of three replicates. Means that do not share the same letters in the column differ obviously at p < 0.05. Bars represent standard deviation.

Under TR4, TR6, TR6L, and TR6S, with DAF, the superior and inferior grain weight of both two cultivars showed a tendency for an increase (**Figure 2**), and the grain-filling rate increased first and then decreased. At 7 DAF, the superior and inferior grain weights in TR4, TR6, TR6L, and TR6S of NS22 were significantly higher than those of NS44. However, at 19, 25, and 31 DAF, the situation reversed, with NS44 significantly higher than NS22 in all treatments. At 7 DAF, under TR6, TR6L, and TR6S, the superior and inferior grain weights and grain-filling rate in RW3 of both cultivars were significantly higher than those in RW1, RW2, TR4RW1, and TR4RW2. Under TR6, the superior and inferior grain weights and grain-filling rate in RW1 of both cultivars were significantly higher than those in RW3 at 19, 25, and 31 DAF. However, under TR6L and TR6S, those in RW3 were significantly higher than those in RW1 (or the difference was not significant) at 7, 13, 19, and 25 DAF. From the dynamic changes in superior grain-filling rate of both cultivars, RW3 was significantly higher than RW1 at 7, 13, 19, and 25 DAF, but significantly lower than RW1 at 31 DAF, and significantly lower than TR4RW1 and TR4RW2, which indicated that TR6L and TR6S may still cause premature senescence of RW3 in the later growth stage. Therefore, it is necessary to further strengthen field management in the later growth stage.

#### 3.2.1 Grain-filling characteristics

Under TR4, there was no significant difference between RW1 and RW2 in the final grain weight, maximum grain-filling rate (MFR), and average grain-filling rate (AFR) of superior and inferior grains of two cultivars (**Table 4**). Under TR6, compared with RW1, the MFR and AFR in RW3 of superior (NS22: 10.19% and 8.33%; NS44: 4.50% and 4.62%) and inferior (NS22: 17.61% and 13.97%; NS44: 4.26% and 5.86%) grains significantly decreased, while after the TR6 was processed to narrow WRHS and add IBS (TR6L and TR6S), the MFR and AFR in RW3 were significantly higher than those in RW1 or had no significant difference. Under TR6L and TR6S, the final grain weight and AFR in RW1 of NS22 were significantly lower than those in TR6RW1, but for NS44, there was no significant difference between RW1 and TR6RW1. Under TR4, TR6, TR6L, and TR6S, the final grain weight and AFR of NS44 were all significantly higher than those of NS22, which may be caused by the later breeding age of NS44 than NS22. Under TR6, compared with RW1, the growth periods in RW3 of both cultivars were significantly reduced by 10.85% and 7.53%, while under TR6L and TR6S, those in RW3 reduced by 6.82%, 6.56%, 3.57%, and 5.04%, respectively.

**Table 4:** Changes in grain-filling characteristics of two cultivars (NS22 and NS44) under four kinds of drip irrigation configurations in 2020 and 2021.

Year	Treatment	Final grain mass (mg)		Maximum grain-filling rate (mg·day^−1^)		Mean grain-filling rate (mg·day^−1^)		Growth period (day)
		Superior	Inferior	Superior	Inferior	Superior	Inferior	
2020	ATR4RW1	47.21 ± 0.328^ef^	40.07 ± 0.684^c^	2.31 ± 0.013^hi^	2.25 ± 0.06^bcd^	1.89 ± 0.0139^ef^	1.60 ± 0.027^c^	87.00 ± 2.000^efgh^
	ATR4RW2	47.49 ± 0.381^e^	40.46 ± 0.338^c^	2.35 ± 0.041^fgh^	2.28 ± 0.078^abcd^	1.90 ± 0.015^e^	1.62 ± 0.014^c^	86.00 ± 2.000^fghi^
	ATR6RW1	46.73 ± 0.128^g^	39.01 ± 0.682^d^	2.32 ± 0.009^hi^	2.15 ± 0.085^gh^	1.87 ± 0.005^g^	1.56 ± 0.027^d^	86.00 ± 1.000^fghi^
	ATR6RW2	45.24 ± 0.22^i^	37.73 ± 0.239^ef^	2.33 ± 0.022^gh^	2.12 ± 0.04^hi^	1.81 ± 0.008^i^	1.51 ± 0.010^ef^	82.33 ± 1.528^klm^
	ATR6RW3	42.84 ± 0.243^j^	33.56 ± 0.152^g^	2.08 ± 0.012^l^	1.77 ± 0.081^j^	1.71 ± 0.010^j^	1.34 ± 0.006^g^	76.67 ± 1.528^p^
	ATR6LRW1	46.21 ± 0.231^h^	38.23 ± 0.596^e^	2.31 ± 0.006^hi^	2.2 ± 0.035^defg^	1.85 ± 0.009^h^	1.53 ± 0.024^e^	88.00 ± 2.000^def^
	ATR6LRW2	47.33 ± 0.349^ef^	40.23 ± 0.306^c^	2.26 ± 0.029^j^	2.12 ± 0.06^h^	1.89 ± 0.014^ef^	1.61 ± 0.012^c^	86.33 ± 0.577^efghi^
	ATR6LRW3	46.36 ± 0.329^h^	38.28 ± 0.61^e^	2.41 ± 0.048^cde^	2.11 ± 0.026^hi^	1.85 ± 0.013^h^	1.53 ± 0.024^e^	82.00 ± 1.000^lmn^
	ATR6SRW1	46.13 ± 0.377^h^	37.57 ± 0.351^f^	2.28 ± 0.017^ij^	2.18 ± 0.04^efgh^	1.85 ± 0.015^h^	1.50 ± 0.014^f^	86.33 ± 1.528^efghi^
	ATR6SRW2	46.18 ± 0.304^h^	39.13 ± 0.546^d^	2.19 ± 0.055^k^	2.05 ± 0.033^i^	1.85 ± 0.012^h^	1.57 ± 0.022^d^	84.67 ± 1.528^hijk^
	ATR6SRW3	47.08 ± 0.058^fg^	39 ± 0.281^d^	2.33 ± 0.036^gh^	2.11 ± 0.02^hi^	1.88 ± 0.002^fg^	1.56 ± 0.011^d^	80.67 ± 1.528^mno^
	BTR4RW1	50.33 ± 0.139^a^	42.77 ± 0.143^a^	2.48 ± 0.027^ab^	2.31 ± 0.004^ab^	2.01 ± 0.006^a^	1.71 ± 0.006^a^	93.00± 1.000^a^
	BTR4RW2	50.32 ± 0.218^a^	42.67 ± 0.145^a^	2.5 ± 0.034^a^	2.3 ± 0.018^abc^	2.01 ± 0.009^a^	1.71 ± 0.006^a^	93.00 ± 1.000^a^
	BTR6RW1	49.61 ± 0.095^bc^	42.65 ± 0.148^a^	2.42 ± 0.017^cd^	2.27 ± 0.014^abcd^	1.98 ± 0.004^bc^	1.71 ± 0.006^a^	93.00 ± 1.000^a^
	BTR6RW2	49.05 ± 0.122^d^	42.12 ± 0.129^ab^	2.39 ± 0.011^def^	2.23 ± 0.006^cdef^	1.96 ± 0.005^d^	1.68 ± 0.005^ab^	90.33 ± 1.528^bcd^
	BTR6RW3	47.32 ± 0.064^ef^	40.15 ± 0.127^c^	2.31 ± 0.021^hi^	2.17 ± 0.033^fgh^	1.89 ± 0.003^ef^	1.61 ± 0.005^c^	86.00 ± 1.000^fghi^
	BTR6LRW1	49.38 ± 0.117^bcd^	42.34 ± 0.14^ab^	2.4 ± 0.019^cde^	2.28 ± 0.016^abc^	1.98 ± 0.005^bcd^	1.69 ± 0.006^ab^	93.33 ± 1.528^a^
	BTR6LRW2	49.17 ± 0.157^d^	42.4 ± 0.279^ab^	2.37 ± 0.02^efg^	2.28 ± 0.029^abc^	1.97 ± 0.006^d^	1.70 ± 0.011^ab^	91.67 ± 0.577^abc^
	BTR6LRW3	49.72 ± 0.039^b^	42.16 ± 0.186^ab^	2.44 ± 0.001^bc^	2.34 ± 0.011^a^	1.99 ± 0.002^b^	1.69 ± 0.007^ab^	90.00 ± 1.000^cd^
	BTR6SRW1	49.24 ± 0.072^cd^	42.24 ± 0.156^ab^	2.4 ± 0.006^cde^	2.28 ± 0.007^abcd^	1.97 ± 0.003^cd^	1.69 ± 0.006^ab^	92.67 ± 1.155^ab^
	BTR6SRW2	49.21 ± 0.044^d^	42.16 ± 0.075^ab^	2.39 ± 0.004^de^	2.26 ± 0.012^abcd^	1.97 ± 0.002^cd^	1.69 ± 0.003^ab^	91.33 ± 1.528^abc^
	BTR6SRW3	49.69 ± 0.092^b^	41.81 ± 0.099^b^	2.43 ± 0.005^cd^	2.32 ± 0.007^ab^	1.99 ± 0.004^b^	1.67 ± 0.004^b^	88.00 ± 0.000^def^
2021	ATR4RW1	47.09 ± 0.329^ef^	39.94 ± 0.691^c^	2.31 ± 0.018^fgh^	2.26 ± 0.057^abc^	1.88 ± 0.013^e^	1.60 ± 0.028^c^	79.33 ± 1.528^efgh^
	ATR4RW2	47.36 ± 0.378^e^	40.33 ± 0.318^c^	2.34 ± 0.042^efg^	2.28 ± 0.08^abc^	1.89 ± 0.015^e^	1.61 ± 0.013^c^	78.67 ± 0.577^fghi^
	ATR6RW1	46.6 ± 0.133^g^	38.89 ± 0.658^d^	2.32 ± 0.008^fgh^	2.15 ± 0.08^ef^	1.86 ± 0.005^fg^	1.56 ± 0.026^d^	77.67 ± 2.082^fghi^
	ATR6RW2	45.11 ± 0.224^i^	37.63 ± 0.222^ef^	2.33 ± 0.023^fgh^	2.11 ± 0.043^f^	1.80 ± 0.009^i^	1.51 ± 0.009^ef^	73.00 ± 1.000^klm^
	ATR6RW3	42.71 ± 0.243^j^	33.43 ± 0.147^g^	2.09 ± 0.011^k^	1.76 ± 0.086^h^	1.71 ± 0.009^j^	1.34 ± 0.006^g^	70.00 ± 1.000^nop^
	ATR6LRW1	46.09 ± 0.225^h^	38.1 ± 0.589^e^	2.3 ± 0.005^ghi^	2.2 ± 0.042^cde^	1.84 ± 0.009^h^	1.52 ± 0.024^e^	78.67 ± 1.528^fghi^
	ATR6LRW2	47.2 ± 0.346^ef^	40.12 ± 0.307^c^	2.25 ± 0.03^i^	2.12 ± 0.061^f^	1.89 ± 0.014^e^	1.60 ± 0.012^c^	78.67 ± 2.082^fghi^
	ATR6LRW3	46.23 ± 0.334^h^	38.15 ± 0.618^e^	2.41 ± 0.046^cd^	2.1 ± 0.028^fg^	1.85 ± 0.013^gh^	1.53 ± 0.025^e^	73.00 ± 1.000^klm^
	ATR6SRW1	46.01 ± 0.382^h^	37.45 ± 0.364^f^	2.28 ± 0.017^hi^	2.18 ± 0.041^def^	1.84 ± 0.015^h^	1.50 ± 0.015^f^	78.67 ± 1.155^fghi^
	ATR6SRW2	46.07 ± 0.301^h^	39 ± 0.545^d^	2.19 ± 0.055^j^	2.03 ± 0.028^g^	1.84 ± 0.012^h^	1.56 ± 0.022^d^	78.00 ± 1.000^fghi^
	ATR6SRW3	46.96 ± 0.064^f^	38.86 ± 0.254^d^	2.34 ± 0.038^fg^	2.11 ± 0.013^f^	1.88 ± 0.003^ef^	1.55 ± 0.010^d^	69.67 ± 1.528^op^
	BTR4RW1	50.21 ± 0.132^a^	42.64 ± 0.138^a^	2.47 ± 0.068^ab^	2.32 ± 0.011^a^	2.01 ± 0.005^a^	1.71 ± 0.006^a^	86.00 ± 2.000^a^
	BTR4RW2	50.19 ± 0.211^a^	42.55 ± 0.147^a^	2.5 ± 0.033^a^	2.29 ± 0.013^ab^	2.01 ± 0.008^a^	1.70 ± 0.006^a^	85.33 ± 0.577^ab^
	BTR6RW1	49.48 ± 0.095^bc^	42.53 ± 0.147^a^	2.42 ± 0.016^cd^	2.28 ± 0.018^abc^	1.98 ± 0.004^bc^	1.70 ± 0.006^a^	84.33 ± 1.155^ab^
	BTR6RW2	48.92 ± 0.123^d^	41.99 ± 0.142^ab^	2.39 ± 0.008^cde^	2.23 ± 0.002^bcd^	1.96 ± 0.005^d^	1.68 ± 0.006^ab^	81.33 ± 1.528^de^
	BTR6RW3	47.19 ± 0.063^ef^	40.03 ± 0.136^c^	2.31 ± 0.02^gh^	2.17 ± 0.046^def^	1.89 ± 0.003^e^	1.60 ± 0.005^c^	75.00 ± 1.000^jk^
	BTR6LRW1	49.27 ± 0.122^bcd^	42.22 ± 0.127^ab^	2.4 ± 0.013^cd^	2.28 ± 0.022^abc^	1.97 ± 0.005^bcd^	1.69 ± 0.005^ab^	85.00 ± 1.000^ab^
	BTR6LRW2	49.05 ± 0.159^d^	42.27 ± 0.271^ab^	2.37 ± 0.021^def^	2.29 ± 0.03^ab^	1.96 ± 0.006^d^	1.70 ± 0.011^ab^	83.67 ± 1.155^abcd^
	BTR6LRW3	49.6 ± 0.04^b^	42.05 ± 0.196^ab^	2.44 ± 0.003^bc^	2.33 ± 0.018^a^	1.98 ± 0.002^b^	1.69 ± 0.008^ab^	78.67 ± 0.577^fghi^
	BTR6SRW1	49.12 ± 0.073^cd^	42.1 ± 0.148^ab^	2.39 ± 0.009^cde^	2.28 ± 0.011^abc^	1.96 ± 0.003^cd^	1.68 ± 0.006^ab^	85.00 ± 0.000^ab^
	BTR6SRW2	49.09 ± 0.049^cd^	42.04 ± 0.091^ab^	2.39 ± 0.002^cde^	2.25 ± 0.019^abc^	1.96 ± 0.002^cd^	1.68 ± 0.004^ab^	83.33 ± 0.577^bcd^
	BTR6SRW3	49.56 ± 0.097^b^	41.68 ± 0.089^b^	2.43 ± 0.002^bc^	2.32 ± 0.006^a^	1.98 ± 0.004^b^	1.67 ± 0.004^b^	76.33 ± 0.577^ij^

A and B represent NS22 and NS44, respectively. TR4 represents a single tube serving four wheat rows under equal row spacing planting, WRHS was 15 cm, DTS was 60 cm; TR6 represents a common single tube serving six wheat rows under equal row spacing planting, DTS was 90 cm, WRHS was 15 cm; TR6L represents an enlarged single tube serving six wheat rows under wide–narrow row planting, DTS was 90 cm, WRHS was 10 cm, IBS was 35 cm; TR6S represents a shortened single tube serving six wheat rows under wide–narrow row planting, DTS was 80 cm, WRHS was 10 cm, IBS was 25 cm. RW1, RW2, and RW3 represent the first, second, and third rows of wheat plants close to the drip tube, respectively. All the values are average of three replicates. Means that do not share the same letters in the column differ obviously at p < 0.05.

### 3.3 Gas exchange

**Figure 3:**
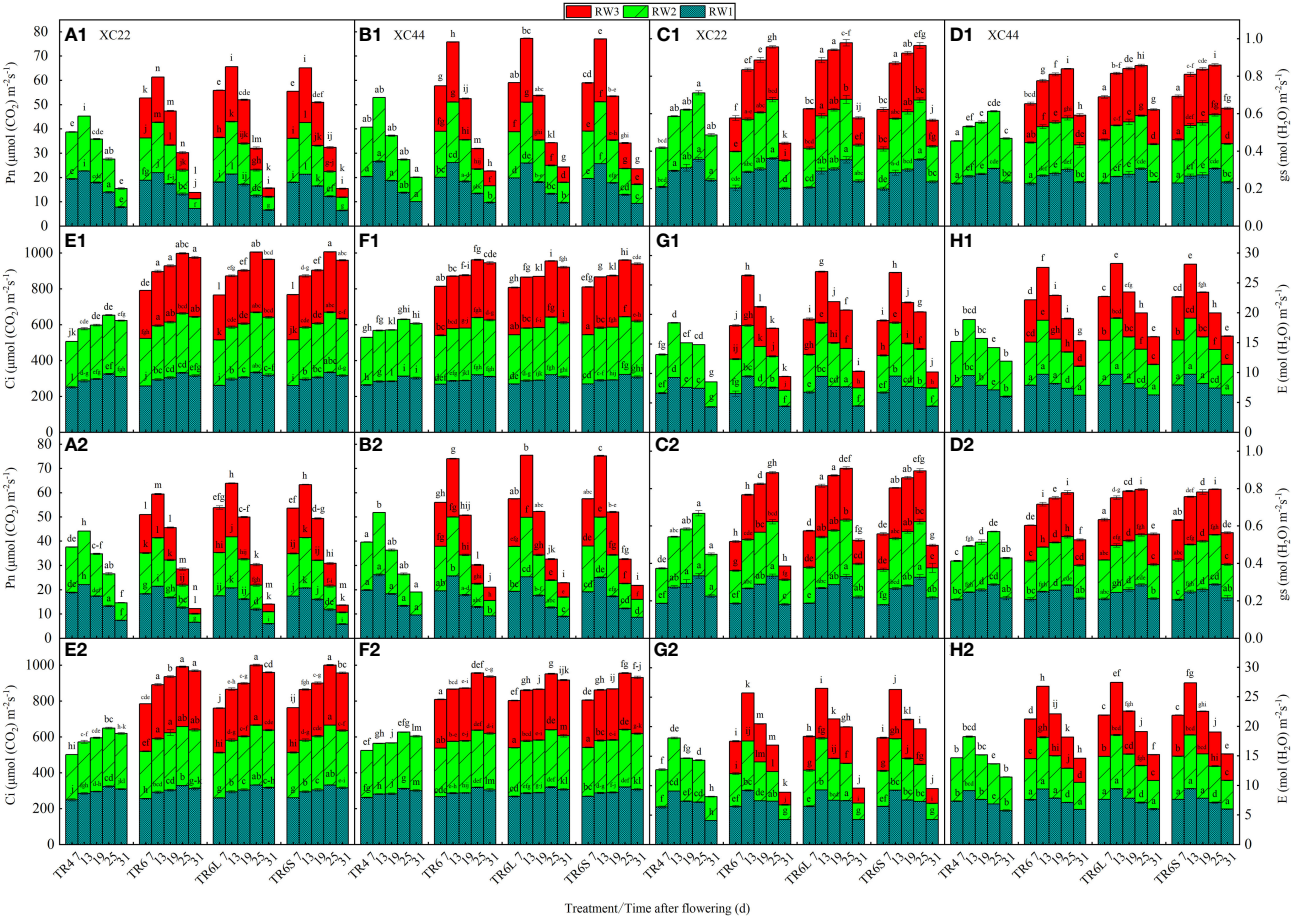
Changes in flag leaf net photosynthetic rate (Pn), stomatal conductance (gs), intercellular CO_2_ concentration (Ci), and transpiration rate (E) with days after flowering of two cultivars (NS22 and NS44) under four kinds of drip irrigation configurations in 2020 and 2021. **(A, B)** represent Pn, **(C, D)** represent gs, **(E, F)** represent Ci, and **(G, H)** represent E. TR4 represents a single tube serving four wheat rows under equal row spacing planting, WRHS was 15 cm, DTS was 60 cm; TR6 represents a common single tube serving six wheat rows under equal row spacing planting, DTS was 90 cm, WRHS was 15 cm; TR6L represents an enlarged single tube serving six wheat rows under wide–narrow row planting, DTS was 90 cm, WRHS was 10 cm, IBS was 35 cm; TR6S represents a shortened single tube serving six wheat rows under wide–narrow row planting, DTS was 80 cm, WRHS was 10 cm, IBS was 25 cm. 1 indicates 2020; 2 indicates 2021. RW1, RW2, and RW3 represent the first, second, and third rows of wheat plants close to the drip tube, respectively. All the values are average of three replicates. Means that do not share the same letters in the column differ obviously at p < 0.05. Bars represent standard deviation.

Under TR4, TR6, TR6L, and TR6S, with DAF, both cultivars showed a tendency for a decrease in the Pn and E, the Ci increased, and the gs increased first and then decreased (**Figure 3**). Under TR6, the Pn, gs, and E of both cultivars showed RW1 > RW2 > RW3, and the Ci showed RW3 > RW2 > RW1. Compared with RW1, at 7, 13, 19, 25, and 31 DAF, the decreases in Pn (6.79%, 5.22%, 7.47%, 37.09%, and 37.27%) and E (9.91%, 8.36%, 9.61%, 23.68%, and 30.77%) in RW3 of NS44 were significantly lower than those of NS22 (Pn: 12.71%, 15.22%, 19.16%, 45.34%, and 63.43%; E: 12.62%, 10.10%, 12.30%, 37.05%, and 46.97%). Under TR6L and TR6S, compared with RW1, the Pn in RW3 of NS22 and NS44 at 7 and 13 DAF significantly increased, while at 25 and 31 DAF, they significantly decreased. The Ci (at 7 and 13 DAF) and E (at 7, 13, 19, 25, and 31 DAF) were significantly lower than those in RW1, and the gs (at 7, 13, and 19 DAF) showed no significant difference compared to those in RW1 indicating that under TR6L and TR6S, the Pn in RW3 during the early and middle grain-filling stages may increase by adjusting E and Ci. However, in the later grain-filling stage, the Pn may still decrease due to insufficient water.

### 3.4 Relative water content and chlorophyll content

**Figure 4:**
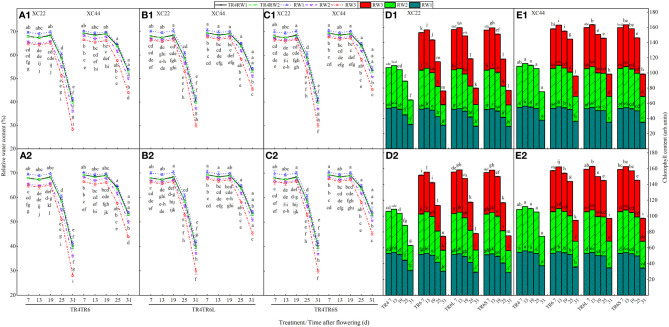
Changes in flag leaf relative water content (RWC) and chlorophyll content (CC) with the days after flowering of two cultivars (NS22 and NS44) under four kinds of drip irrigation configurations in 2020 and 2021. **(A–C)** represent RWC; **(D, E)** represent CC. TR4 represents a single tube serving four wheat rows under equal row spacing planting, WRHS was 15 cm, DTS was 60 cm; TR6 represents a common single tube serving six wheat rows under equal row spacing planting, DTS was 90 cm, WRHS was 15 cm; TR6L represents an enlarged single tube serving six wheat rows under wide–narrow row planting, DTS was 90 cm, WRHS was 10 cm, IBS was 35 cm; TR6S represents a shortened single tube serving six wheat rows under wide–narrow row planting, DTS was 80 cm, WRHS was 10 cm, IBS was 25 cm. 1 indicates 2020; 2 indicates 2021. RW1, RW2, and RW3 represent the first, second, and third rows of wheat plants close to the drip tube, respectively. All the values are average of three replicates. Means that do not share the same letters in the column differ obviously at p < 0.05. Bars represent standard deviation.

Under TR4, TR6, TR6L, and TR6S, with DAF, the relative water content (RWC) and chlorophyll content (CC) in the flag leaves of both NS22 and NS44 gradually decreased, and the decrease was slow in the early stage and sharp in the later stage (**Figure 4**). Under TR4, the RWC (7, 25, and 31 DAF) and CC (7, 13, 19, 25, and 31 DAF) of NS44 were significantly higher than those of NS22. Under TR6, the RWC and CC of the two cultivars showed RW1 > RW2 > RW3. Compared with that of RW1, the RWC in RW3 at 7, 13, 19, 25, and 31 DAF significantly decreased by 5.16%–31.74%, and the CC significantly decreased by 2.25%~41.94%. After the TR6 was processed to narrow WRHS and add IBS (TR6L and TR6S), the RWC and CC in RW3 of both cultivars were significantly higher than those in TR6RW3 at 7, 13, 19, 25, and 31 DAF. The CCs in RW3 of both cultivars were significantly higher than those in RW1 at 7 and 13 DAF, while at 25 and 31 DAF, they were significantly lower than those in RW1. This was probably due to the rise in temperature at the late grain-filling stage (the rise in soil moisture evaporation caused by IBS), which deteriorated the growth environment of RW3 wheat plant.

### 3.5 Flag leaf area

**Figure 5:**
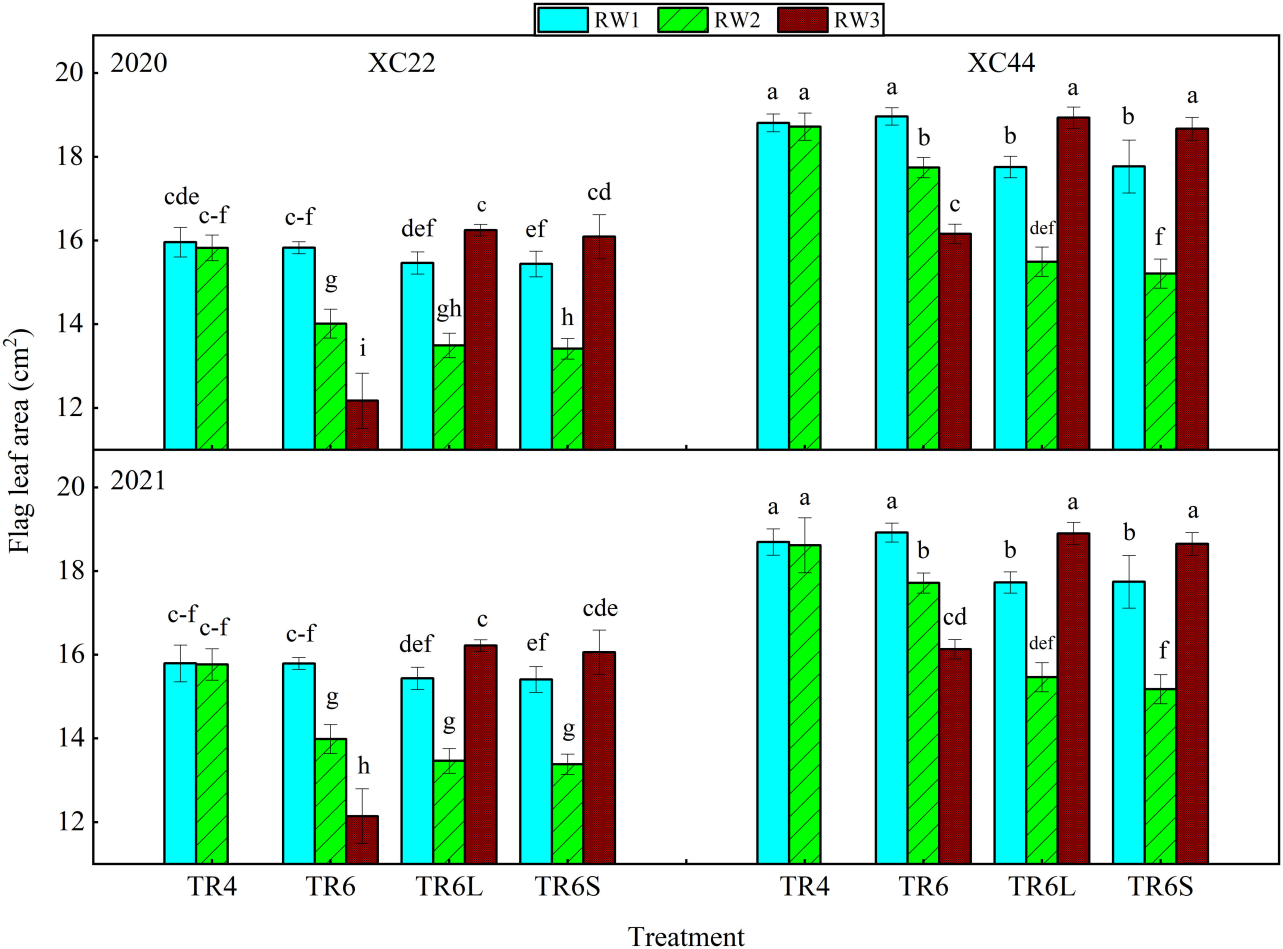
Changes in flag leaf area of two cultivars (NS22 and NS44) under four kinds of drip irrigation configurations in 2020 and 2021. TR4 represents a single tube serving four wheat rows under equal row spacing planting, WRHS was 15 cm, DTS was 60 cm; TR6 represents a common single tube serving six wheat rows under equal row spacing planting, DTS was 90 cm, WRHS was 15 cm; TR6L represents an enlarged single tube serving six wheat rows under wide–narrow row planting, DTS was 90 cm, WRHS was 10 cm, IBS was 35 cm; TR6S represents a shortened single tube serving six wheat rows under wide–narrow row planting, DTS was 80 cm, WRHS was 10 cm, IBS was 25 cm. 1 indicates 2020; 2 indicates 2021. RW1, RW2, and RW3 represent the first, second, and third rows of wheat plants close to the drip tube, respectively. All the values are average of three replicates. Means that do not share the same letters in the column differ obviously at p < 0.05. Bars represent standard deviation.

Under TR4, the flag leaf area (FLA) in RW1 exhibited no significant distinction from RW2 for both cultivars (**Figure 5**), the FLA of NS44 was significantly larger than that of NS22. Under TR6, the FLA of both NS22 and NS44 showed RW1 > RW2 > RW3. Compared with RW1, the FLA decrease in RW3 of NS22 (23.09%) was significantly higher than that of NS44 (14.80%). After the TR6 was processed to narrow WRHS and add IBS (TR6L and TR6S), the FLA of both NS22 and NS44 showed RW3 > RW1 > RW2. Compared with RW1, the FLAs in RW3 of NS22 significantly increased by 5.05% and 4.21%, and those of NS44 increased by 6.63% and 5.04%. It was worth mentioning that compared with TR6RW2, the FLA in RW2 of NS22 and NS44 under TR6L and TR6S decreased, indicating that the flag leaf development of RW2 may be inhibited due to insufficient ventilation and light transmission after narrowing the row spacing, The decreases of NS44 (4.28% and 14.28%) were greater than those of NS22 (3.73% and 12.68%), which were probably related to the fact that NS44 had a stronger environmental adaptability.

### 3.6 Chlorophyll fluorescence parameters

**Figure 6:**
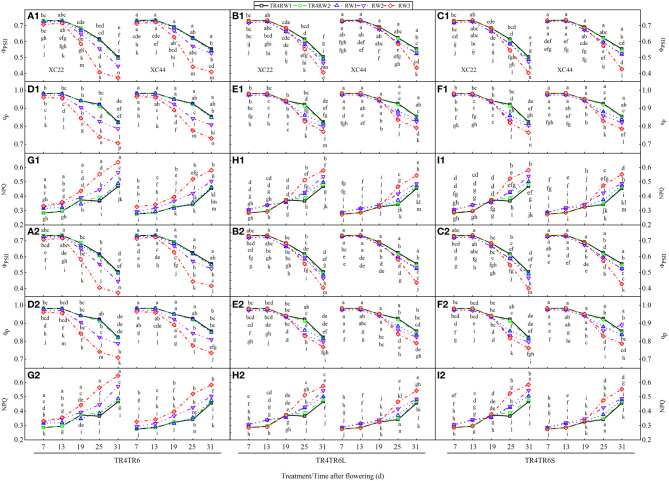
Changes in actual photochemical efficiency of PSII (ΦPSII), photochemical quenching coefficient (qp), and non-photochemical quenching coefficient (NPQ) of flag leaf with the days after flowering of two cultivars (NS22 and NS44) under four kinds of drip irrigation configurations in 2020 and 2021. **(A–C)** represent ΦPSII; **(D–F)** represent qp; **(G–I)** represent NPQ. TR4 represents a single tube serving four wheat rows under equal row spacing planting, WRHS was 15 cm, DTS was 60 cm; TR6 represents a common single tube serving six wheat rows under equal row spacing planting, DTS was 90 cm, WRHS was 15 cm; TR6L represents an enlarged single tube serving six wheat rows under wide–narrow row planting, DTS was 90 cm, WRHS was 10 cm, IBS was 35 cm; TR6S represents a shortened single tube serving six wheat rows under wide–narrow row planting, DTS was 80 cm, WRHS was 10 cm, IBS was 25 cm. 1 indicates 2020; 2 indicates 2021. RW1, RW2, and RW3 represent the first, second, and third rows of wheat plants close to the drip tube, respectively. All the values are average of three replicates. Means that do not share the same letters in the column differ obviously at p < 0.05. Bars represent standard deviation.

Under TR4, TR6, TR6L, and TR6S, with DAF, the actual photochemical efficiency of PSII (ΦPSII) and photochemical quenching coefficient (qp) of both NS22 and NS44 showed a trend to decline, and the decline was slow in the early period and sharp in the later period (**Figure 6**). The non-photochemical quenching coefficient (NPQ) showed a trend to increase. Under TR6, at 7, 13, 19, 25, and 31 DAF, the ΦPSII and qp of both NS22 and NS44 showed RW1 > RW2 > RW3, while the NPQ showed RW3 > RW2 > RW1.Compared with RW1, the ΦPSII and qp in RW3 of NS22 and NS44 significantly decreased by 1.19%–33.35%, and the NPQ significantly increased by 5.52%–49.09%. After the TR6 was processed to narrow WHRS and add IBS (TR6L and TR6S), at 7, 13, 19, 25, and 31 DAF, the ΦPSII and qp in RW3 of both NS22 and NS44 were significantly higher than those in TR6RW3, and the NPQ was significantly lower than that of TR6RW3. At 7, 13, and 19 DAF, the ΦPSII and qp in RW3 were higher than those in RW1 and lower than those in RW1 at 25 and 31 DAF, which may be related to the fact that the inter-block near RW3 could bring marginal advantage in the early grain-filling stage, while in the late grain-filling stage, it would cause deterioration of the wheat plant growth environment.

## 4 Discussion

### 4.1 Effects of different drip irrigation configurations on yield traits and economic benefit

Scarcity of fresh water resources has seriously affected wheat production in the arid and semiarid areas (Anwaar et al., 2019; Shen et al., 2013). one promising solution is to develop high irrigation efficiency systems, such as surface drip irrigation, which could save approximately one-third of fresh water annually compared to flood irrigation (Wang et al., 2018; Yang D et al., 2020). In Xinjiang (northwest region of China), surface drip irrigation systems have been successfully applied to the production of crash crops, such as cotton (Wang et al., 2019) and corn (Guo Q et al., 2021), since 1996, and in 2008, mature crash crop drip irrigation systems were first applied to wheat production (Chen et al., 2015). Due to the limitation on horizontal movement distance of water, a single tube serving four rows of wheat (row space was 15 cm) configuration (TR4, two wheat rows on each side of the drip tube) was established and popularized. However, in recent years, due to the continuous increase in the price of the drip tube, the disadvantage of TR4 requiring a large amount of drip irrigation tubes (high investment) has become increasingly prominent, and the development of wheat drip irrigation system encountered new challenges (Lv et al., 2019). Changing the ratio of drip tube to wheat row and adjusting wheat row spacing may be a new way to increase drip-irrigated wheat yield and economic benefits (Lv et al., 2019; Wan et al., 2022). In this study, it was found that under TR4, the grain income and economic benefits of NS44 (3,199.27 and 2,211.50 US$ ha^−1^) were significantly higher than those of NS22 (3,024.80 and 2,037.03 US$ ha^−1^), which was consistent with the research results of Yang JP et al. (2020) and Wan et al. (2022). Further analysis found that the panicle numbers in RW1 and RW2 of NS44 were significantly less than those of NS22 by 17.25% and 17.12%, while the GWP (19.77% and 19.80%), TGW (21.44% and 20.69%), growth period (6.90% and 8.14%), and AGFR of superior (6.61% and 5.95%) and inferior (6.72% and 5.47%) grains were significantly higher than those of NS22. These suggested that the yield strategies of NS22 and NS44 may differ under the TR4 system. NS22’s yield relied more on the population quantity (number of panicles), whereas NS44 depended more on individual quality (GWP). Consequently, it can be inferred that wheat varieties, such as NS44 (with higher GWP and lower tillers), could be better suited for planting with drip irrigation systems. After increasing the DTS from 60 to 90 cm under 15-cm equal WRHS condition (TR6), the yields of NS22 and NS44 were significantly decreased by 9.93% and 9.04%, and the superior (10.60% and 4.89%) and inferior (23.19% and 7.78%) grain weight per panicle and GWP (13.95% and 5.76%) were significantly decreased. These indicated that under TR6, the yield decrease of NS22 was higher than that of NS44, which was related to the decrease in GWP, especially in inferior grain. This was consistent with the result of previous research that there are variety differences in the adaptability of wheat to changes in growth environment (Yang et al., 2022), and inferior grain was more sensitive to environmental factors (Luo et al., 2019). The TGW of NS44 (6.62%) decreased more than that of NS22 (3.55%), which may be related to the smaller decrease in the number of inferior grains per panicle compared to that of NS22. This further indicated that to clarify the grain weight of wheat, it was necessary to analyze superior and inferior grains separately (Jiang et al., 2003). From the results of GWP (superior and inferior) and yield in RW1, RW2, and RW3 under TR6, both NS22 and NS44 showed RW1 > RW2 > RW3, and the difference was significant. Compared with RW1, the GWP, panicle number, and yield in RW3 of NS22 and NS44 decreased by 19.75%, 9.00%, 18.39%, 9.77%, 4.57%, and 16.68% respectively. Therefore, we inferred that the yield decrease in RW3 was the main reason for the decrease in yield under TR6, which was consistent with previous research (Wan et al., 2022). After TR6 was processed to narrow WHRS and add 35-cm IBS (TR6L), the GWP of NS22 and NS44 compared with TR6 increased by 6.80% and 1.59%, and the GWP and yield in RW3 were significantly higher than those of TR6RW3 by 26.05%, 19.64%, 10.50%, and 20.35%. However, the yields of TR6L were still lower than that of TR4 by 9.88% and 5.35%. In this situation, we determined that the 35-cm IBS may be too wasteful of land. After shortening IBS from 35 to 25 cm (TR6S), the yield of NS22 and NS44 compared with those of TR6 increased by 7.24% and 8.79%, and the economic benefits were higher than those of TR4, TR6, and TR6L by 3.18%, 11.39%, 9.12%, 5.47%, 11.50%, and 3.86%, and the results were consistent between 2020 and 2021. It was worth noting that the GWPs in RW1 of both NS22 and NS44 under TR6S were significantly lower than those of TR6RW1 and TR4RW1, which was probably due to the deficiency in photosynthetic radiation caused by the 10-cm WHRS. For both NS22 and NS44, the panicle numbers of RW1 and RW2 under TR6S were significantly lower than those of TR4, TR6, and TR6L, which was probably due to inadequate sowing density per row resulting from the rise in the number of wheat rows within a given unit of area. Thus, we believed that the TR6S should continue being optimized by improving photosynthetic radiation of RW1 (adopting slope planting, as shown in **Supplementary Figure 3**) and appropriately increasing the sowing density.

### 4.2 Effects of different drip irrigation configurations on photosynthetic physiology of flag leaves and grain filling

**Figure 7:**
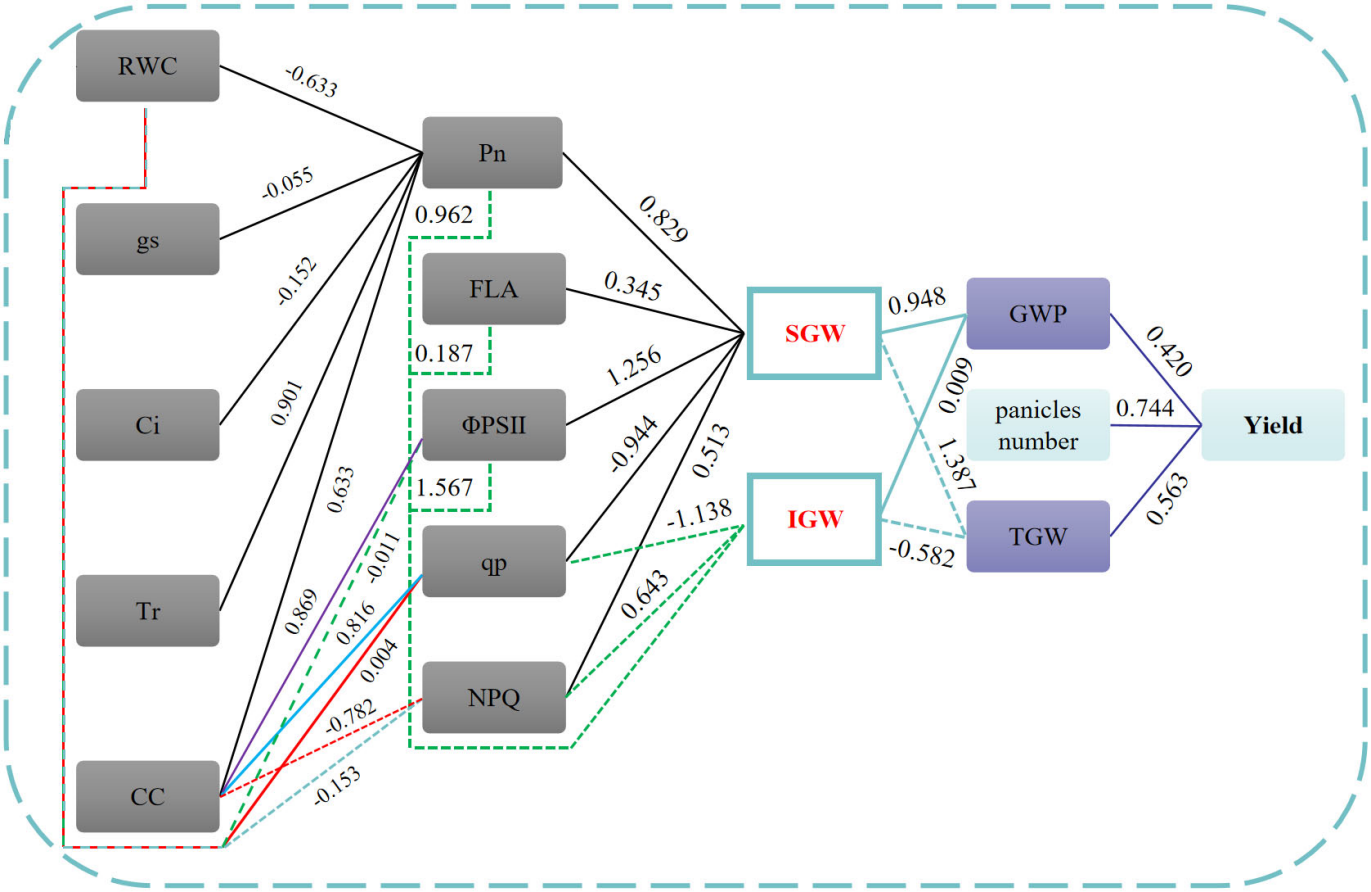
Path analysis of all test factors on wheat yield. SGW and IGW represent superior and inferior grain weights, respectively. All numbers represent the direct path coefficient.

The yield potential of wheat is divided into the following three major components: the panicle number per unit area, the grain number per panicle, and the grain weight (Yu, 2013). As the final yield component, grain weight is mainly determined by grain-filling rate and period (Brocklehurst, 1977; Yang and Zhang, 2006). Flag leaf is the most important photosynthetic organ in the process of grain filling (Evans and Rawson, 1970), and the photosynthetic physiological processes of the flag leaf are very sensitive to soil water content (Kang et al., 2016). Studying the changes in grain weight of superior and inferior grains and photosynthetic physiology of the flag leaf with DAF under different DTS and WRHS conditions has important implications for clarifying the formation mechanism of drip-irrigated wheat grain yield and would provide optimization suggestions for establishing lower-cost wheat drip irrigation system. In this study, it was found that under TR4, the FLA of both NS22 and NS44 showed no significant difference between RW1 and RW2, and the FLA of NS44 was significantly bigger than that of NS22 by 18.07%, which may be the direct reason NS44 has a higher GWP compared with NS22. This was consistent with previous research suggesting that under abiotic stress conditions, developed source organs play an important role in coping and adapting to the environment, which, in turn, increase crop yield (Gui et al., 2021). After increasing the DTS from 60 to 90 cm under a 15-cm equal WRHS condition (TR6), the FLA of both NS22 and NS44 showed that RW1 > RW2 > RW3. The difference was significant, which was consistent with the results of previous studies that there was a significant difference in the leaf area index between rows of wheat under an enlarged drip irrigation system (Chen et al., 2015). At the same time, this study also found that after the TR6 was processed to narrow WRHS and add IBS (TR6L and TR6S), the FLA in RW3 of both NS22 and NS44 significantly increased, while the FLA in RW1 decreased. This indicated that flag leaf growth was very sensitive to environmental changes. Thus, we inferred that the size of the FLA could be used to simply determine the quality of the wheat growth environment during the booting stage. For both NS22 and NS44, there was no significant difference between RW1 and RW2 in the final superior and inferior grain weight, AFR, and growth period under TR4, while after increasing the DTS from 60 to 90 cm under a 15-cm equal WRHS condition (TR6), the final superior and inferior grain weights of NS22 and NS44 decreased by 5.11%, 8.67%, 2.51%, and 3.31%, respectively. The decrease in NS44 was lower than that in NS22, and both NS22 and NS44 showed greater influence on inferior grain than on superior grain. Under TR6, TR6L, and TR6S, the superior and inferior grain weights and grain-filling rate in RW3 of both NS22 and NS44 at 7 DAF were significantly higher than those in RW1, TR4RW1, and TR4RW2, which was possibly due to the inappropriate growth environment of RW3 before flowering, and the environment led to an increase in transportation rate of dry matter stored in vegetative organs to grains, which was consistent with previous studies (Yang et al., 2001). Under TR6, for both of NS22 and NS44, the Pn, E, RWC, CC, ΦPSII, and qp of the flag leaf in RW3 at 7, 13, 19, 25, and 31 DAF were significantly lower than those in RW1, TR4RW1, and TR4RW2, and the gap between RW3 and RW1 increased with the grain-filling process indicating that under a single tube serving six wheat rows configuration, the flag leaf photosynthesis of RW3 was restricted by the environment during the whole grain-filling period, and the restriction was intensified with the grain-filling process. After TR6 was processed to narrow WHRS and add IBS (TR6L and TR6S), at 7, 13, 19, 25, and 31 DAF, the Pn of NS22 and NS44 under TR6L increased by 6.01%, 6.98%, 9.75%, 6.05%, 12.49%, 2.36%, 1.94%, 2.46%, 7.54%, and 7.38%, respectively, and under TR6S, they increased by 5.15%, 6.19%, 7.58%, 7.17%, 11.09%, 2.10%, 1.60%, 1.88%, 7.13%, and 3.92% respectively. At the same time, the E, RWC, CC, ΦPSII, and qp under TR6L and TR6S all showed varying degrees of increase, and the Ci and NPQ all showed varying degrees of decrease. These indicated that an enlarged drip irrigation system using a wide–narrow row configuration to plant wheat could improve the photosynthetic physiological status of flag leaves, which may be related to the improvement of soil water and fertilizer caused by a narrow wheat row and the marginal advantage of plant growth brought by a wide wheat row. Based on the results of this study, the possible regulatory model of a drip irrigation pattern on wheat grain yield was proposed (**Figure 7**). From the results of Pn in RW1, RW2, and RW3 under TR6L and TR6S, for both NS22 and NS44, the Pn values of RW3 were significantly higher than those of TR6RW3 at 7, 13, 19, 25, and 31 DAF. Compared with RW1, at 7, 13, and 19 DAF, the Pn values in RW3 of NS22 (TR6L: 7.64%, 5.51%, and 1.47%; TR6S: 5.95%, 2.18%, and 1.74%) and NS44 (TR6L: 7.42%, 8.50% and 0.86%; TR6S: 5.87%, 1.80% and 1.70%) significantly increased, while at 25 and 31 DAF, the Pn values of RW3 (NS22: 28.07%, 45.35%, 29.49%, and 33.70%; NS44: 18.77%, 44.61%, 18.49%, and 31.55%) significantly decreased. These indicated that TR6L and TR6S could improve the photosynthetic physiology of RW3 in the early grain-filling stage, while in the late grain-filling stage, the photo-system of the flag leaf may still be damaged due to unsuitable growth environments, which may be due to the rise in temperature during the late grain-filling stage (IBS induced an increase in potential soil moisture evaporation). Thus, we believe that the TR6S should continue being optimized by adjusting irrigation and fertilization strategies (time and amount) to ensure that the demands of different rows of plants are adequately met.

## 5 Conclusions

After increasing the DTS from 60 (TR4) to 90 cm (TR6) under a 15-cm equal WRHS condition, the yield of both NS22 (9.93%) and NS44 (9.04%) decreased significantly. The primary cause for the reduction in yield can be attributed to the decrease in GWP of RW3. The decrease in GWP was caused by the reduced grain-filling rate, and a decrease in inferior grain was higher than that of superior grain. The decline in grain-filling rate was related to the decrease in FLA, Pn, CC, RWC, ΦPSII, and qp of R3. After TR6 was processed to narrow WHRS (from 15 to 10 cm) and add IBS (TR6L: 35 cm and TR6S: 25 cm), the AFR of superior and inferior grains in RW3 of both NS22 and NS44 significantly increased. Among TR4, TR6, TR6L, and TR6S, for both NS22 and NS44, the yields of TR6S were significantly higher than those of TR6 and TR6L, and both cultivars showed the highest economic benefits under TR6S. Based on the results of this study, we recommend the TR6S to be further optimized by improving the photosynthetic radiation of RW1 appropriately increasing sowing density and adjusting irrigation and fertilization strategy.

## Data availability statement

The original contributions presented in the study are included in the article/**Supplementary Material**. Further inquiries can be directed to the corresponding author.

## Author contributions

JJ: Formal analysis, Investigation, Software, Writing – original draft, Writing – review & editing. FQ: Data curation, Formal analysis, Investigation, Software, Writing – original draft, Writing – review & editing. ZL: Investigation, Resources, Writing – original draft. XC: Investigation, Writing – original draft. WL: Data curation, Project administration, Supervision, Writing – original draft, Writing – review & editing.
