# Supplementary material for: Narrowing row spacing and adding inter-block promote the grain filling and flag leaf photosynthetic rate of wheat under enlarged drip tube spacing system
Source: Front Plant Sci. 2024 Jun 5;15:1368410. doi: 10.3389/fpls.2024.1368410 (PMC11188436; doi:10.3389/fpls.2024.1368410)
Supplement: Supplementary file 2 [file DataSheet_1.zip › Supplementary information/Supplementary Figure 2.pdf]

XC22 TR4 (at flowering stage)

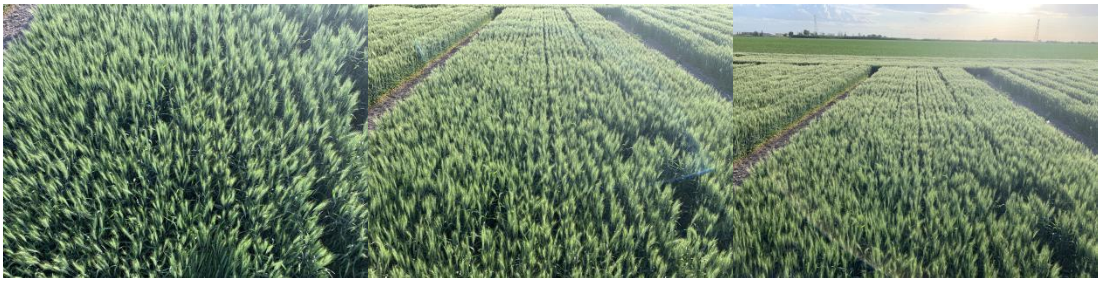

XC22 TR6 (at booting stage)

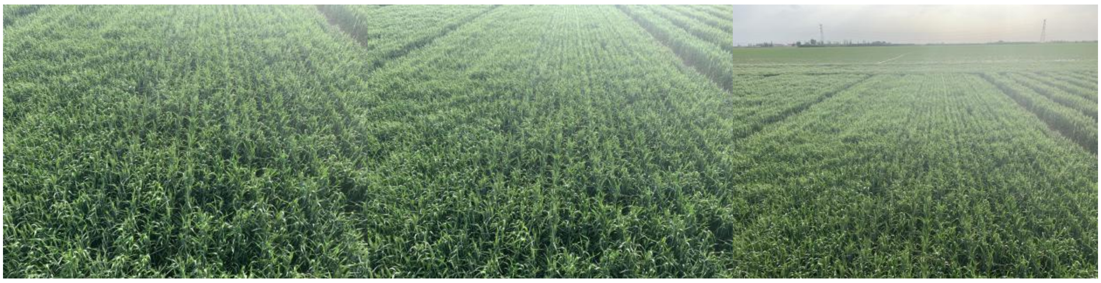

XC22 TR6L (at booting stage)

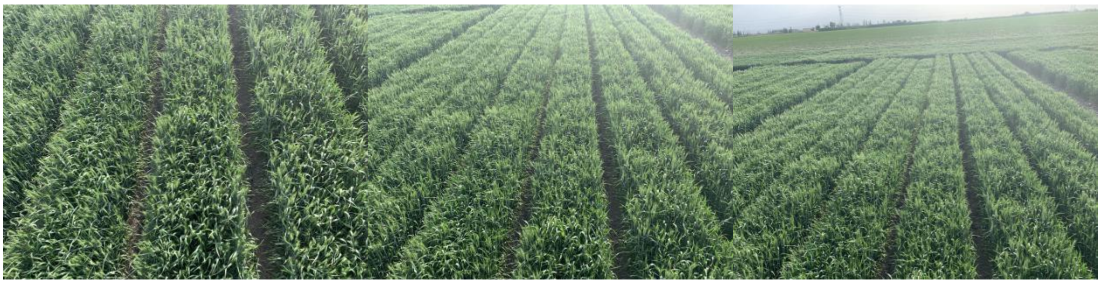

XC22 TR6S (at flowering stage)

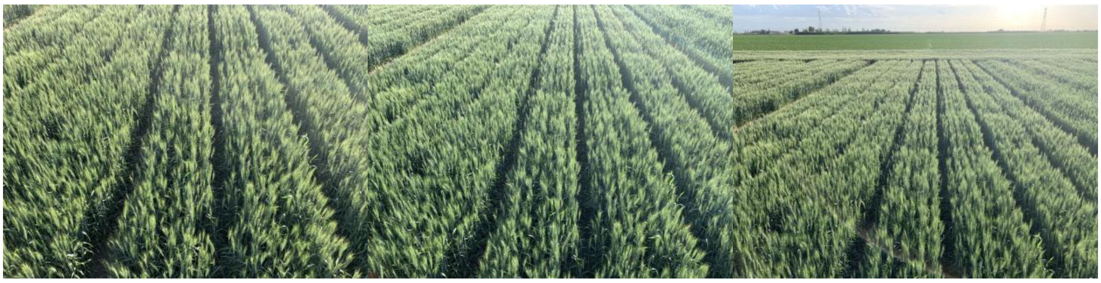

XC44 TR4 (at booting stage)

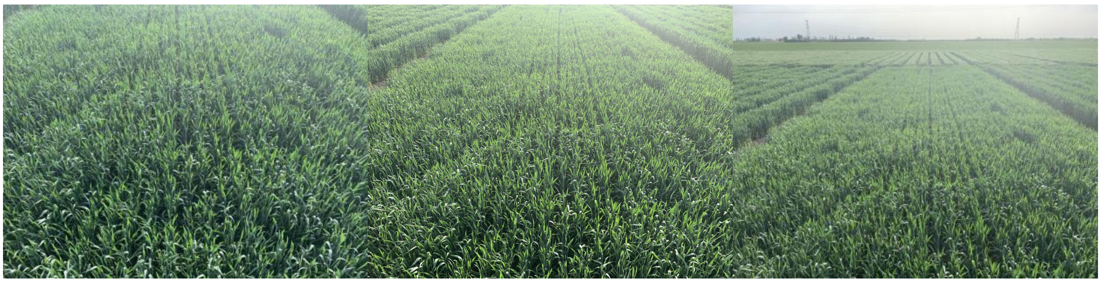

XC44 TR6 (at flowering stage)

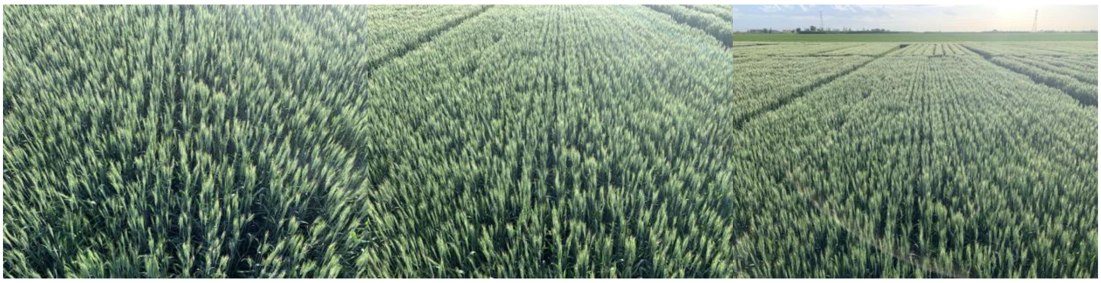

XC44 TR6L (at booting stage)

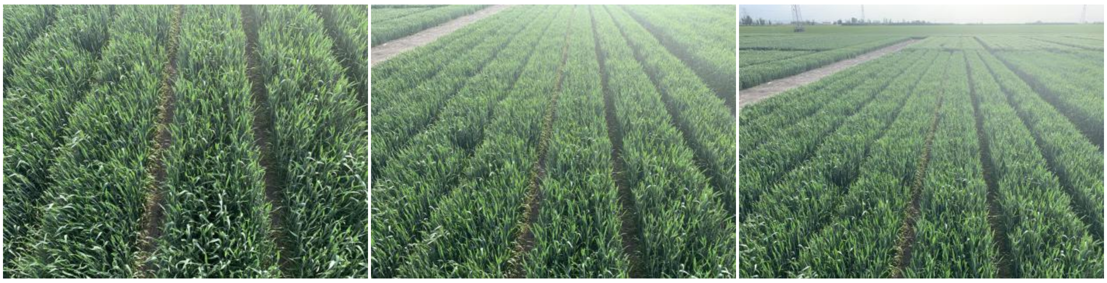

XC44 TR6S (at booting stage)

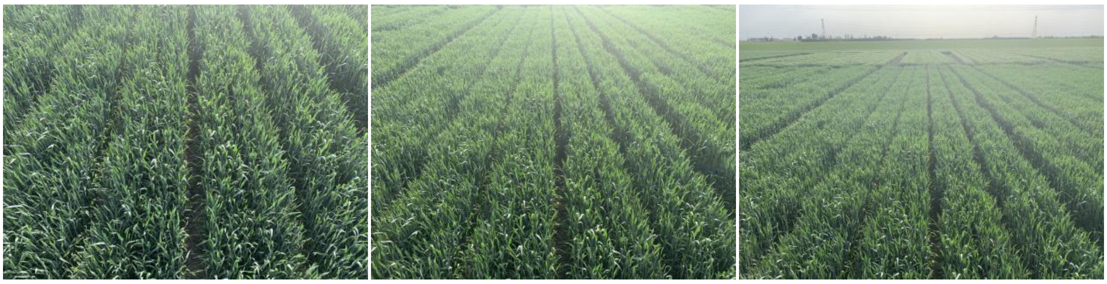

Figure S1. Photos of plant growth in the field
