# Supplementary figures and images for: Narrowing row spacing and adding inter-block promote the grain filling and flag leaf photosynthetic rate of wheat under enlarged drip tube spacing system
Source: Front Plant Sci. 2024 Jun 5;15:1368410. doi: 10.3389/fpls.2024.1368410 (PMC11188436; doi:10.3389/fpls.2024.1368410)

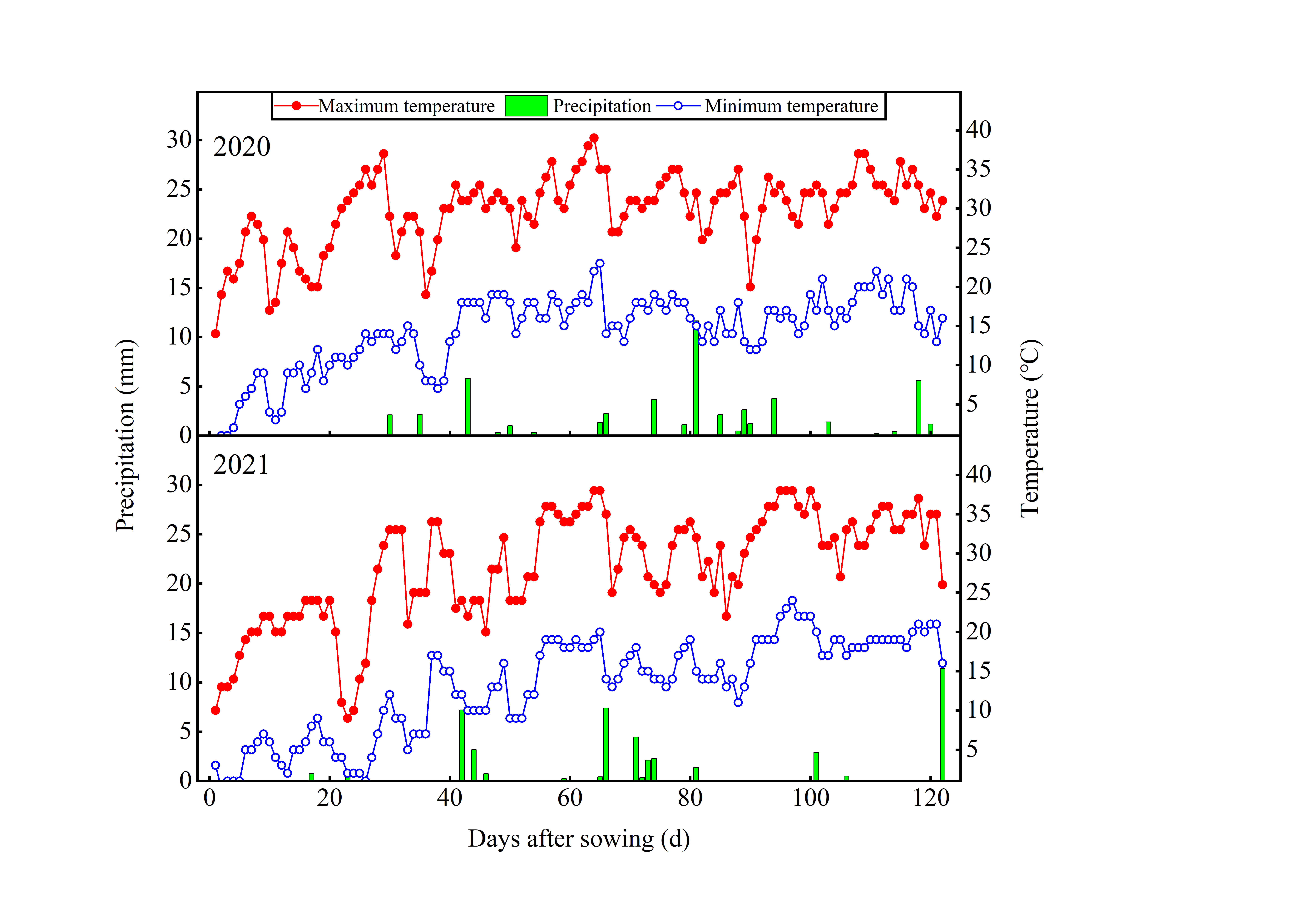

Supplement: Supplementary file 2 [file DataSheet_1.zip › Supplementary information/Supplementary Figure 1.tif]
