# Supplementary material for: Narrowing row spacing and adding inter-block promote the grain filling and flag leaf photosynthetic rate of wheat under enlarged drip tube spacing system
Source: Front Plant Sci. 2024 Jun 5;15:1368410. doi: 10.3389/fpls.2024.1368410 (PMC11188436; doi:10.3389/fpls.2024.1368410)
Supplement: Supplementary file 2 [file Supplementary Figure 3.pdf › Supplementary information/Supplementary Figure 3.pdf]

A

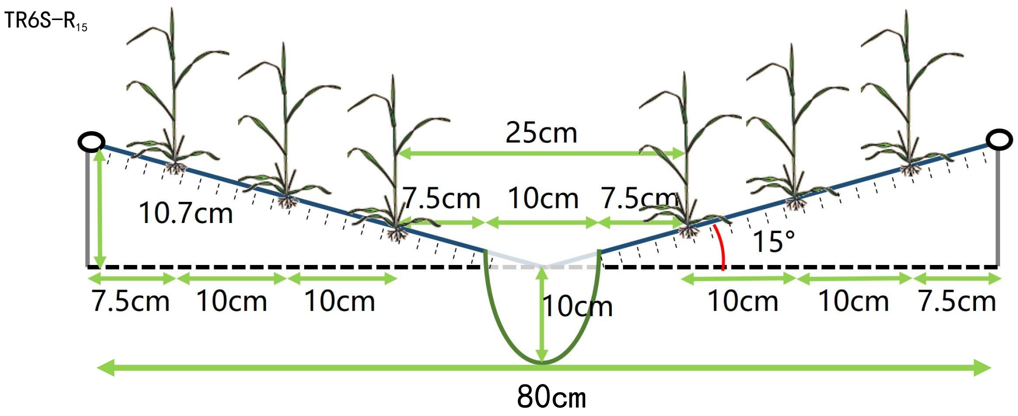

B

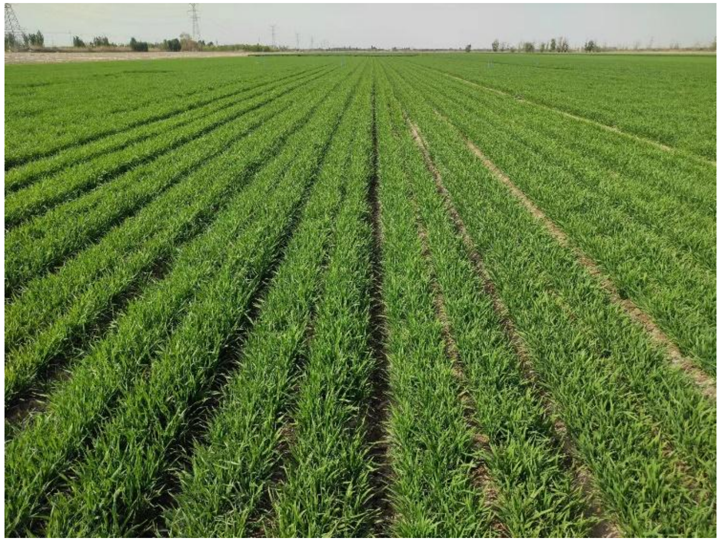

Figure S2. Schematic diagram of slope drip irrigation pattern. A: Schematic diagram; B: Pre experimental field photos
